# Insights into the cell-wall dynamics in grapevine berries during ripening and in response to biotic and abiotic stresses

**DOI:** 10.1007/s11103-024-01437-w

**Published:** 2024-04-11

**Authors:** Giulia Malacarne, Jorge Lagreze, Barbara Rojas San Martin, Mickael Malnoy, Marco Moretto, Claudio Moser, Lorenza Dalla Costa

**Affiliations:** 1 Research and Innovation Centre, Fondazione Edmund Mach, San Michele all’Adige, 38098 Trento, Italy; 2 Centre Agriculture Food Environment (C3A), University of Trento, San Michele all’Adige, 38098 Trento, Italy

**Keywords:** Cell wall, Grapevine, Berry development, Texture, *Botrytis cinerea*, Drought

## Abstract

The cell wall (CW) is the dynamic structure of a plant cell, acting as a barrier against biotic and abiotic stresses. In grape berries, the modifications of pulp and skin CW during softening ensure flexibility during cell expansion and determine the final berry texture. In addition, the CW of grape berry skin is of fundamental importance for winemaking, controlling secondary metabolite extractability. Grapevine varieties with contrasting CW characteristics generally respond differently to biotic and abiotic stresses. In the context of climate change, it is important to investigate the CW dynamics occurring upon different stresses, to define new adaptation strategies. This review summarizes the molecular mechanisms underlying CW modifications during grapevine berry fruit ripening, plant-pathogen interaction, or in response to environmental stresses, also considering the most recently published transcriptomic data. Furthermore, perspectives of new biotechnological approaches aiming at modifying the CW properties based on other crops’ examples are also presented.

**Supplementary Information:**

The online version contains supplementary material available at 10.1007/s11103-024-01437-w.

## Key message

we provide a comprehensive overview of the mechanisms behind cell wall modeling in grapevine during berry development and environmental stresses, with a focus on biotech applications in fleshy fruit trees.

**Supplementary Information:**

The online version contains supplementary material available at 10.1007/s11103-024-01437-w.

## General introduction

### Overview of plant cell wall (CW) components

Primary cell walls (PCWs) are heterogeneous structures across different species, tissues, and developmental stages and have established roles in maintaining and determining cell shape, resisting internal turgor pressure, directing cell and plant growth, contributing to plant morphology, and regulating diffusion through the apoplast (Swaminathan et al. 2022). Cell expansion requires rapid synthesis and extensive remodeling of CW material. In some specific cases, mature cells produce a secondary and much thicker CW to ensure water and solute transport through the vasculature and mechanical support of the plant (Schuetz et al. 2013). The two CW types are referred to as primary and secondary CW (PCWs and SCWs) (Gilbert 2010; Li et al. 2016; Anderson and Kieber 2020).

The PCW is a complex structure located outside the plasma membrane and has been extensively reviewed in the literature (Carpita and Gibeaut 1993; Caffal and Mohnen 2009; Cosgrove 2016; Cosgrove 2023). In dicotyledons, the PCW, referred to as type 1 PCW, is composed predominantly (∼ 90%) of polysaccharides from three major classes (cellulose, hemicelluloses, and pectic polysaccharides) and, to a lesser extent of structural proteins and phenolics, minerals, and enzymes (∼ 10%) (Carpita and Gibeaut 1993). It is generally accepted that each polysaccharide component makes up an equal proportion of the CW, approximately one-third of the dry weight each, although this proportion may vary according to species, cell and tissue type, developmental and environmental context (Goulao et al. 2012).

The cellulose is arranged in long and stiff microfibrils, composed of 1, 4-β-D-glucan chains, linked by extensive hydrogen bonds, which are largely parallel but interlaced, analogous to the structural arrangement of individual threads in a cotton fabric. They provide most of the tensile strength to the plant cell wall and are embedded in a complex matrix consisting of hemicelluloses and pectic polysaccharides. The hemicellulose consists of neutral glycans that interact non-covalently through hydrogen bonds with the cellulose microfibrils to form an extensive backbone. In type 1 PCW, the most abundant hemicellulose is xyloglucan, a neutral polysaccharide composed of a 1, 4-β-D-glucan backbone that differs from cellulose by having numerous regularly spaced xylose side chains (Carpita and Gibeaut 1993).

Pectins (homogalacturonan, xylogalacturonan, rhamnogalacturonan I, and rhamnogalacturonan II) are embedded in the cellulose/hemicellulose network, forming a hydrophilic gel. They are acidic polysaccharides, enriched in galacturonic acid residues, and can be linear or branched. Homogalacturonan (HG), the most abundant pectin, consists of continuous α-1,4-linked galacturonic acid residues, which can be methyl esterified at the C6 carboxyl groups and acetylated at O^2^ and O^3^ positions. Rhamnogalacturonan I (RG-I) has a backbone of alternating galacturonic acid and rhamnose residues with large linear or branched arabinan and galactan side chains (Brummell 2006). Rhamnogalacturonan II (RG II), a highly conserved and complex pectin, has an HG backbone decorated with side chains containing 13 different sugar subunits and over 20 different glycosyl linkages. Hemicelluloses and pectins are matrix polysaccharides. The structural diversity of pectin is due both to its complex biosynthetic process, which requires at least 67 different transferases, including glycosyltransferases, methyltransferases, and acetyltransferases (Atmodjo et al. 2013), and to post-synthetic processes of polysaccharide assembly and remodeling (Bellincampi et al. 2014).

The middle lamella is a pectin-rich layer between neighboring cells that provides intercellular junctions and dissolves as the fruit ripens (Jarvis et al. 2003).

The much thicker and stronger SCW, which accounts for most of the carbohydrates in biomass, is synthesized when the cell stops dividing and expanding. In fact, in some tissues, lignin is deposited within the cellulose microfibrils, replacing pectin molecules, forming bonds with non-cellulosic carbohydrates, and creating a thick SCW. Lignin is a hydrophobic polyphenolic compound composed of monolignol subunits that are covalently cross-linked by laccases and CW peroxidases (Vanholme et al. 2010). Lignin can be covalently linked to the ferulate side chains of xylans (Swaminathan et al. 2022). In contrast to PCWs, which are relatively elastic due to effective CW remodeling, SCWs are characterized by high stiffness (Donaldson 2001). The formation of SCWs occurs mainly in xylem vessels, structural fibers, seed pods and integuments (Bonawitz and Chapple 2010). Indeed, most fleshy fruits are composed mainly of parenchyma cells with only a thin PCW. In contrast, a few fruits (e.g. pear [*Pyrus* spp.] and loquat [*Eriobotrya Japonica*]) contain lignified cells with SCWs. For this reason, only changes in PCW will be considered in this review.

### PCW dynamics in fleshy fruits during ripening

During the ripening process of fleshy fruits, a series of structural and compositional changes occur in the networks of polymers that forms the PCW, generally changing the characteristics of the unripe fruit with a hard texture to the ripe and attractive fruit with a soft texture (Payasi et al. 2009). The loss of firmness (a process known as softening) makes ripe fruit more susceptible to environmental stresses and postharvest decay (Kuchi and Sharavani 2019). Programmed softening, which occurs during the early ripening of many fruit species, involves the progressive dissolution of the xyloglucan–cellulose network, leading to CW loosening and disassembly of pectin resulting in the dissolution of the middle lamella (Jarvis et al. 2003; Brummell 2006; Mercado et al. 2011; Paniagua et al. 2017; Posé et al. 2019).

Fruit softening is a developmentally regulated process, and most of the modifications in CW polymers occurs by tight genetic controls of the Cell Wall Modifying Enzymes (CWMEs), which rely on secretory pathways. Indeed, due to the complex composition and spatial structure of CW carbohydrates (Caffal and Mohnen 2009), many CWMEs are involved in CW modifications during fruit ripening (Table 1). These enzymes make specific contributions to the softening process (Brummell and Harpster 2001; Brummell 2006) and are interdependent at the same time. For example, HG is initially synthesized in the Golgi in a highly methyl esterified form and is then de-esterified at the cell wall level by pectin methylesterases (PMEs), making it the appropriate substrate for polygalacturonases (PGs) (Brummell and Harpster 2001). Moreover, expansins (EXP) are required to increase the accessibility of PG to the substrate (Cantu et al. 2008a; Jiang et al. 2019). Finally, although pectate lyases (PLs) are responsible for the loss of de-esterified HG (pectin backbone) at the tricellular junction (TCJ) and middle lamella (ML) (Wang et al. 2019), it appears that additional PG activity is required for the complete depolymerisation of homogalacturonan (Ortega-Salazar et al. 2023).

**Table 1:** Main cell wall modifying enzymes (CWMEs) involved in CW modification, their mode of action, and their substrate

CWME enzyme	Mode of action	Substrate
Pectin methylesterase (PME) EC 3.1.1.11	Removal of methyl groups from methyl-esterified pectin	Methyl-esterified pectin
Pectate lyase (PL) EC 4.2.2.2.	Cleavage of unesterified pectin by a β-elimination reaction	Unesterified pectin
Endo-polygalacturonase (PG) EC 3.2.1.15	Hydrolysis of the a-1,4-glucuronide links in homogalacturonan	Unesterified pectin
Expansin (EXP) EC 4.2.2.10	Disruption of non-covalent interactions between hemicelluloses and cellulose microfibrils	Cellulose, hemicellulose
Xyloglucan-endotransglucosylase/hydrolase (XTH) EC 2.4.1.207	Hydrolysis and/or transglycosylation of xyloglucan terminal	Hemicellulose
Rhamnogalacturonan lyase (RGL) EC 4.2.2.23	Hydrolysis of the α-1,2 linkages between galacturonosyl and rhamnosyl residues in pectin	Unesterified pectin
Cellulase (CEL) EC 3.2.1.4	Hydrolysis of β-1,4 glucan linkages in cellulose and xyloglucan	Cellulose, hemicellulose
β-galactosidase (β-gal) EC 3.2.1.23	Terminal removal of galactosyl residues from pectin	Unesterified pectin

Many of these enzymes have been identified in many fruit crops, most of them also in grapevine, as transcribed by multigene families, due to their multiple roles in different plant development processes and responses to biotic and abiotic stresses. Table 1 lists the main CWDEs involved in CW modification during fruit ripening, together with their mechanism of action and substrate.

### PCW involvement in the response to biotic and abiotic stresses during fruit ripening

Fruit ripening, which is characterized by biophysical, physiological, transcriptional, and biochemical changes, is often associated with a significant increase in susceptibility to fungal pathogens, resulting in large economic losses of perishable horticultural products. (Giovannoni 2001; Cantu et al. 2008a; Alkan and Fortes 2015; Blanco-Ulate et al. 2015). Multiple changes have been identified in the tomato–*B. cinerea* pathosystem, which has emerged as a model for fruit–necrotrophic interactions (Cantu et al. 2009; Petrasch et al. 2019; Silva et al. 2021).

**Fig. 1:**
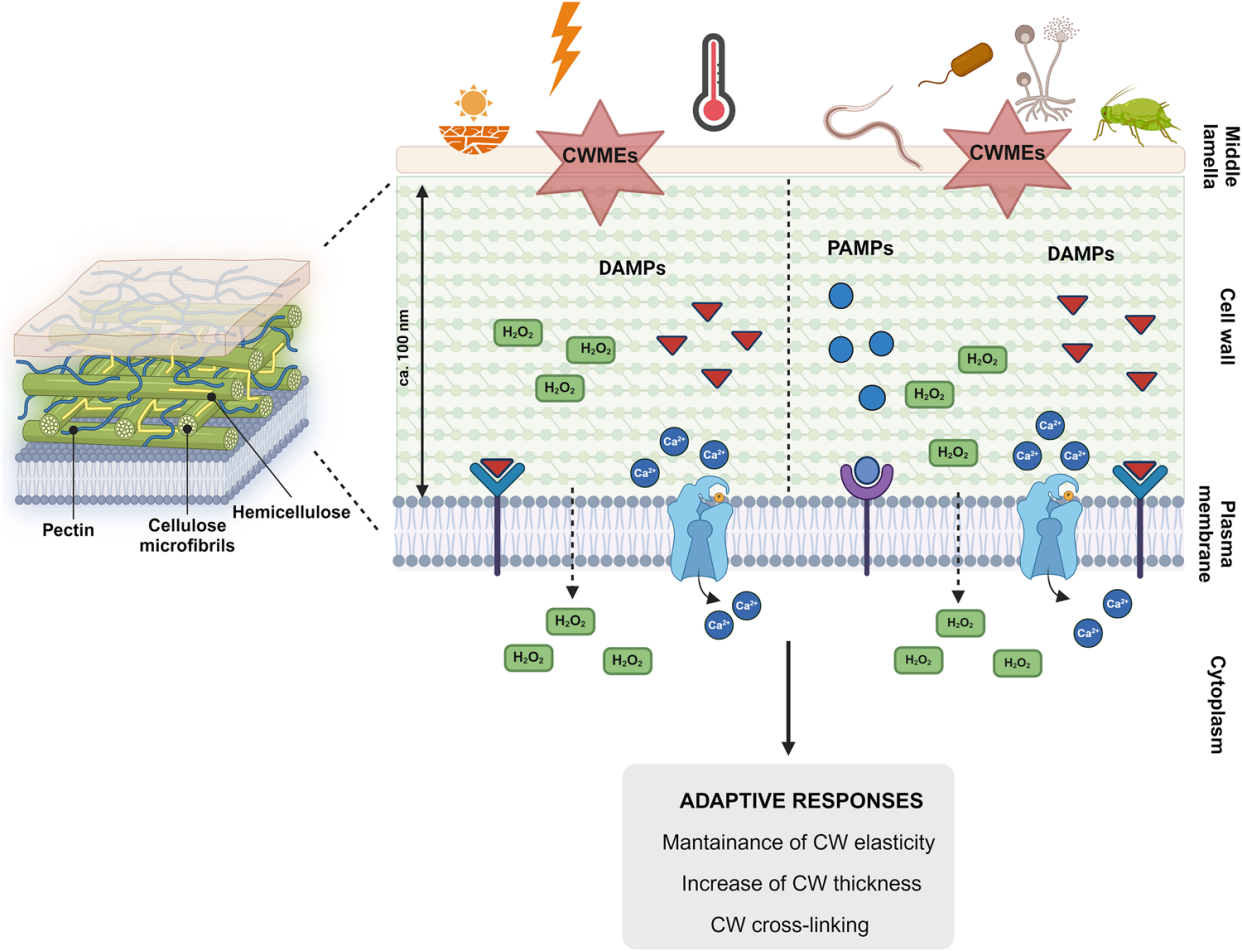
Overview of Cell Wall Integrity (CWI) maintenance system to keep the functional integrity of the CW upon biotic (right side) and abiotic (left side) stresses. The main adaptive responses implemented by the plant upon recognition of DAMPs (red triangles) and PAMPs (blue circles) by plasma membrane receptors are indicated. During this process, changes in calcium (Ca^2+^) and reactive oxygen species (ROS) levels, mainly H_2_O_2_, occur. DAMPs (Damage-Associated Molecular Patterns), PAMPs (Pathogen-Associated Molecular Patterns), and CWMEs (Cell Wall Modifying Enzymes). The artwork was created using BioRender.com

To counteract CW dissolution upon fungal attack or other environmental stresses, plant cells have evolved a Cell Wall Integrity (CWI) maintenance system, the function of which is to maintain the functional integrity of the CW during biotic and abiotic stresses (Vaahtera et al. 2019; Baez et al. 2022; Swaminathan et al. 2022). Plant cells possess plasma membrane sensors and pattern recognition receptors (PRRs) to sense pathogen-associated molecular patterns (PAMPs) and damage-associated molecular patterns (DAMPs) and, upon recognition, initiate an adaptive response to maintain CWI (Fig. 1). DAMPs include fragments of degraded cell wall polymers, such as oligogalacturonides (OGs), derived from pectin degradation and associated with changes in calcium (Ca^2+^) and reactive oxygen species (ROS) levels (Vaahtera et al. 2019). Plant laccases and cell wall peroxidases (PRXs) may regulate ROS levels by scavenging H_2_O_2_ for cross-linking reactions within the cell wall, thereby affecting CW strength and stiffness (Swaminathan et al. 2022). Disease-tolerant genotypes activate the CWI system more strongly than the susceptible genotypes. Moreover, the level of susceptibility/tolerance of a plant to different pathogens depends on the action of specific CWMEs and specific members of the CWI system, whose activation/repression is highly regulated at the transcriptional level and is genotype-dependent (Engelsdorf et al. 2018; Vaahtera et al. 2019).

A ripe fruit, characterized by a less rigid CW, also becomes more susceptible to abiotic stresses, such as water stress caused by drought, light stress (quality and quantity), high temperature, and high salinity. The effects of different abiotic stresses on primary and secondary CW metabolism have been intensively studied (see Table 1 in Le Gall et al. 2015). In general, it is difficult to summarize a common pattern of adaptive responses to the different abiotic stresses in plants, as the overall effects depend on the plant species and the genotype, the age of the plant, the time of stress application, and its intensity. However, two main processes alter CW properties to counteract environmental stress: (i) the increase in CW elasticity (CWE) mainly due to the activation of specific xyloglucan endotransglucosylase/hydrolase (XTH) and expansin (EXP) proteins that remodel the CW architecture; (ii) CW thickening due to a massive deposition of cellulose and hemicellulose in the PCW (Le Gall et al. 2015) (Fig. 1).

The second part of this review focuses on the grapevine, namely on the CW changes that occur during berry ripening and under biotic and abiotic stresses, highlighting the genes likely to be responsible for these processes. In addition, an overview is given of the studies carried out in many fleshy fruit crops to functionally characterize the main CWME-encoding genes involved in fruit softening using biotechnological approaches.

## The case of grapevine

### CW modifications during grapevine berry development and influence on berry texture

Grapevine (*Vitis* spp.) is a widely cultivated and economically important fruit crop comprising more than 50 species, used to produce table grapes, raisins, and wine (Vivier and Pretorius 2000). However, almost all the world’s wine and grapes for fresh consumption are produced from only one of them, *Vitis vinifera L.*, which is native to the southern area of the Caucasus Mountains and the Caspian Sea (Jay 1996).

Textural characteristics of grape berries depend on the grape cultivar and phytohormone treatments (Peppi et al. 2006; Rojas et al. 2021) and are critical in determining consumer appreciation in the case of table grapes, and polyphenol extractability in the case of wine grapes (Ortega-Regules et al. 2006).

The pericarp of grape berries includes the mesocarp (flesh), which consists of cells with thin cell walls, and the exocarp (skin), which contains thick-walled epidermal and subdermal cells with abundant plastids and polyphenols (Hardie et al. 1996). Differences in CW composition between the skin and the flesh (Ortega-Regules et al. 2008) and between the epidermis and hypodermis of berry skin were reported (Fasoli et al. 2016). The berry skin plays a very important role in regulating berry growth and softening through its CW remodeling (Huang et al. 2005; Schlosser et al. 2008), and it has been argued that skin loosening triggers flesh loosening, which contributes to whole berry softening (Huang and Huang 2001; Vicens et al. 2009).

**Fig. 2:**
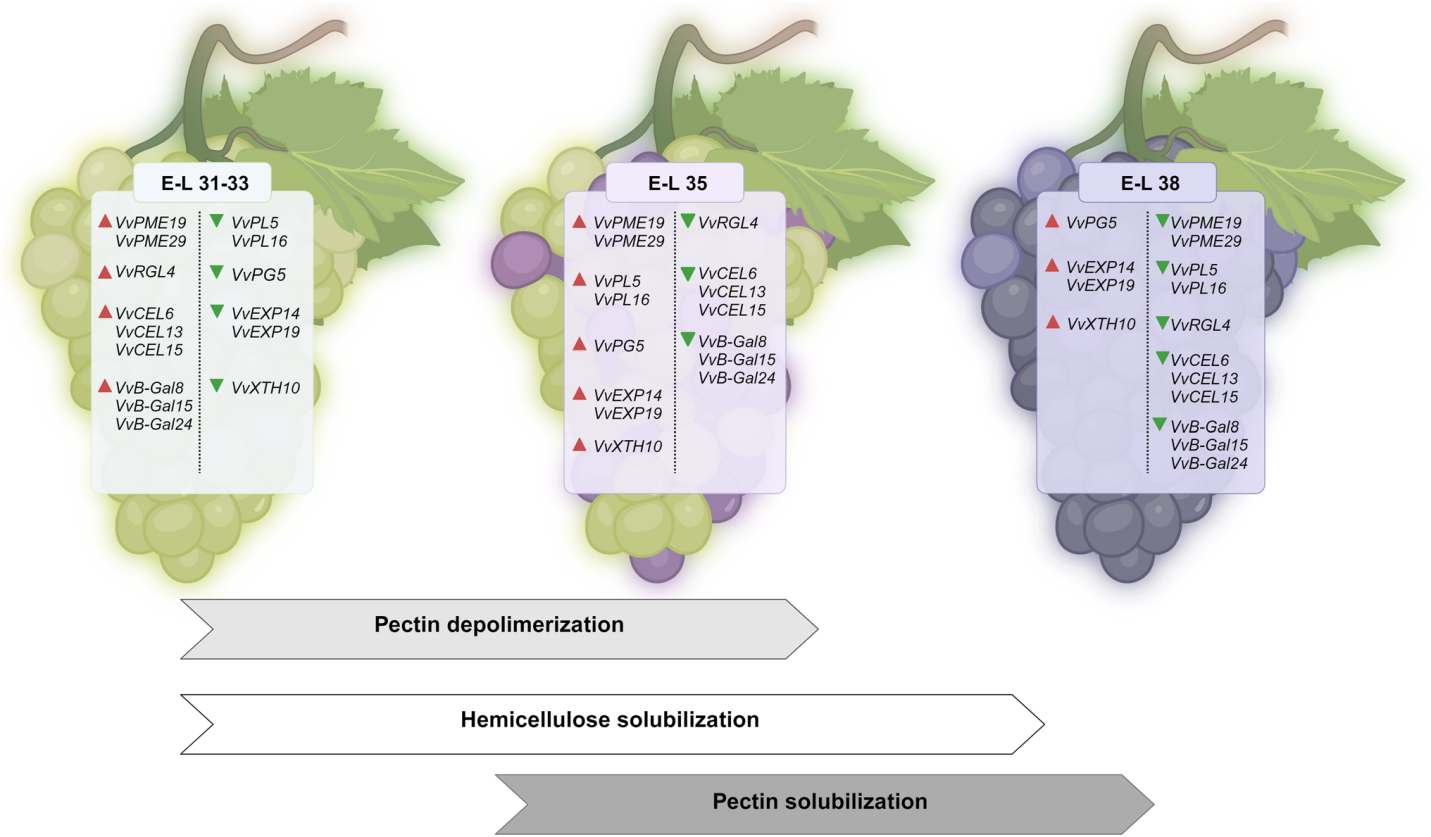
Schematic representation of CW modifications during berry development with the most modulated CWME-encoding gene members. Data refers to a transcriptional study on ‘Pinot Noir’ cultivar (Fasoli et al. 2018). For the abbreviations of the CWMEs, refer to Table 1. Red triangles: up-regulation; green triangles: down-regulation. The artwork was created using BioRender.com

The biochemical variations of CW affect its viscosity and porosity, determining berry texture during development (Ruiz-May and Rose 2013), and correspond to a modulation of the expression of CW-related genes (Grimplet et al. 2007; Zenoni et al. 2010; Dal Santo et al. 2013). The transcript profiles of CWMEs are dynamic as shown in Fig. 2, which summarizes the main CW modifications that occur during berry development along with the most modulated CWME-encoding gene members in a transcriptional study on ‘Pinot Noir’ (Fasoli et al. 2018). This wine grape variety was chosen as a model for soft berries to highlight the genes that are most closely associated with the softening process.

The growth of grape berries follows a double sigmoid pattern, characterized by a lag phase between two growth phases (Coombe and McCarthy 2000; Conde et al. 2007). During the first growth phase, precisely between E-L 31 and E-L 33, a high rate of cell division in the pericarp tissue occurs. At this stage, the main CW modifications consist in pectin depolymerization by means of pectin methylesterase (PME), rhamnogalacturonan lyase (RGL) and β-galactosidase (β-Gal) and in the loosening of the xyloglucan-cellulose network by the action of cellulase (CEL) with consequent hemicellulose solubilization. In this phase the most up-regulated gene members in ‘Pinot Noir’ are *VvPME19* (VIT_12s0035g01900^1^) and *VvPME29* (VIT_09s0002g00330), *VvRGL4* (VIT_18s0001g07850), *VvCEL6* (VIT_02s0025g01380), cellulases VvCEL13 (VIT_07s0005g00740), and VvCEL15 (VIT_12s0035g02180) and *Vv-β-Gal8* (VIT_04s0023g02690) *Vv-β-Gal15* (VIT_09s0002g02120), *Vv-β-Gal24* (VIT_18s0001g13230) (Fig. 2).

The next phase, the lag phase, coincides with the véraison (E-L 35) and is characterized by the absence of changes in weight and volume of the berry and by the onset of berry coloration. During this phase, the softening process is characterized by pectin solubilization due to the activity of PMEs, PGs and PLs, among others. Pectin de-methyl esterification by PMEs is a critical step in berry development, and the regulation of PME activity can be also controlled by PME inhibitors (PMEIs), as in the case of the *VvPMEI1* gene identified as being involved in grape berry development (Lionetti et al. 2015). Notably, the degradation of the pectin in the middle lamella with the loss of intercellular adhesion is the main phenomenon inducing fruit softening. As reported in Fig. 2, the most up-regulated genes in this phase in ‘Pinot Noir’ are *VvPME19* (VIT_12s0035g01900) and *VvPME29* (VIT_09s0002g00330), *VvPL5* (VIT_05s0051g00590) and *VvPL16* (VIT_17s0000g09810), and *VvPG5* (VIT_08s0007g08330), *VvEXP14* (VIT_13s0067g02930), *VvEXP19* (VIT_18s0001g01130) and *VvXTH10* (VIT_06s0061g00550). As an alternative process to the hydrolysis by PGs and PLs, de-esterified pectin can also interact with calcium ions (Ca^2+^) to form the so-called “egg box” motif, resulting in an increased cell wall stiffness. Several works have shown a correlation between calcium content and berry firmness in grapevines, with firmer cultivars usually showing higher calcium content in the cell wall (Balic et al. 2014; Ejsmentewicz et al. 2015; Rojas et al. 2021).

The second growth phase is characterized by a decrease in CW stiffness and by cell expansion due to increased turgor pressure. This phase also marks the onset of berry ripening (E-L 38), when the berry accumulates sugars and anthocyanins in red cultivars (Coombe 1976). At the level of gene regulation, specific members of PG, EXP, and XTH gene families are kept up-regulated (Fig. 2).

Compared to table grapes, which are generally firmer than wine grapes, the main differences concern the *PME*, *PG*, and *XTH* gene classes. It is worth noting that in the table grape ‘Gordo’, *VvPME19* (VIT_12s0035g01900) was stably expressed during the berry development, without major variations (Nunan et al. 2001), while in the variety ‘Muscat Hamburg’, *VvPME6* (VIT_11s0016g00290) is the most up-regulated gene member (Ma et al. 2020). In the same variety, *VvPG5* was down-regulated during ripening (Ma et al. 2020). Interestingly, *VvXTH10* did not show a strong up-regulation in the firm table grape variety ‘Red Globe’ (Ma et al. 2020). On the other hand, a common network between wine and table grape varieties can be observed at the *PL* gene level, as *VvPL5* and *VvPL16* are also the most expressed genes in ‘Thompson Seedless’ and ‘Muscat Hamburg’ at véraison (Balic et al. 2018; Ma et al. 2020, respectively). In addition, *VvPL16* was found to be highly expressed also in the table grape ‘Kyoho’ at véraison (Ma et al. 2023). Such evidence indicates *VvPL16* as one of the most important *PL* genes involved in berry softening.

### CW modifications in grapevine berries showing berry shrivel symptoms

Berry shrivel (BS) is one of the most prominent and still not fully understood physiological disorders of grape ripening, affecting grapevine yield and berry quality (Savoi et al. 2022). The symptoms are visible after véraison and consist of a significant reduction in sugar accumulation, enhanced content of organic acids, low pH values, and, in red varieties, reduced biosynthesis of anthocyanins in berry skins (Savoi et al. 2019). In a recent study, Savoi et al. (2019, 2022) performed a transcriptome analysis of rachis and berries from asymptomatic and symptomatic clusters, highlighting substantial transcriptional changes associated with CW modification and degradation in BS-symptomatic berries. Genes encoding cellulose synthases and xyloglucan endotransglucosylase showed lower expression in the rachis, similar to what was observed during sugar starvation. In contrast, genes related to CW modification and degradation, i.e. *VvEXPA6* (VIT_06s0004g04860), *XTH32* (VIT_06s0061g00550), *BXL1* (VIT_05s0077g01280), and *VvPME3* (VIT_09s0002g00320), were induced, attesting an enhanced CW relaxation, which could facilitate the access of hydrolytic enzymes to degrade the CW polymers (Savoi et al. 2022). The same authors found that, in general, in berries at véraison, before BS visible symptoms, CWME-encoding genes were expressed at a low level, while later, during berry ripening and in symptomatic berries, the same genes were strongly modulated, either enhanced or repressed (Savoi et al. 2019). Whether these CW modifications in the rachis and the berry in the BS symptomatic plants are a cause or a consequence of the disorder is still to be determined.

### CW modifications during berry ripening in response to *Botrytiscinerea*

Variation in CW composition during berry ripening correlates with increased susceptibility to pathogens, particularly the necrotrophic fungus *Botrytis cinerea* (*Bc*) (Weiller et al. 2021). *Bc* is one of the most important pathogens affecting grapevine berries during ripening, causing bunch rot disease, which is most observed on ripe berries following rainfalls or a long period of high humidity close to harvest (Williamson et al. 2007). During infection, *Bc* secretes an array of pectin backbone-modifying enzymes, hemicellulose-modifying proteins, that can target pectin and hemicellulose side branches, and enzymes predicted to degrade cellulose. All these fungal CWMEs are essential virulence factors for the *Botrytis* infection process (Blanco-Ulate et al. 2014). Indeed, *Bc* can modify, disrupt, and degrade the pectin networks of berry fruit through the action of its PGs and PMEs (L’enfant et al. 2015; Li et al. 2022).

Several studies have aimed to decipher the interaction between different *Vitis* genetic backgrounds and *Bc* at the molecular level, by analyzing metabolites and transcripts. At the metabolite level, the variation in CW composition of grapes during ripening (Moore et al. 2014; Tian et al. 2019), in agreement with the recent results of Weiller et al. (2021), could partially explain the difference in susceptibility of grapevines to *Bc*, particularly between véraison and ripe stages. Moreover, Weiller et al. (2021) highlighted that berries from table grape cultivars, which appeared to be more susceptible to *Bc* than wine grape berries, also exhibited corresponding pectin de-methylesterification and depolymerization, glucan production, and extensive glycoprotein deposition. In addition, André et al. (2021) studied different parameters in ‘Pinot Noir’ and ‘Chardonnay’ berries (the former showing more susceptibility to *Bc*) and identified a correlation between the skin thickness and composition with the different levels of susceptibility to *Bc*. At the transcript level, an important focus was the dual transcriptome analysis of grape berries and *Bc*, highlighting the genes modulated in both the host and the fungal pathogen during their interaction. Kelloniemi et al. (2015) provided an integrated view of fungal and grapevine berry molecular events upon *Bc* infection in the susceptible cultivar ‘Marselan’. Many *Bc* genes up-regulated upon infection of mature berries are involved in the degradation of the plant CW. Furthermore, Agudelo-Romero et al. (2015) and Haile et al. (2017, 2020) showed that in the susceptible cultivars ‘Trincadeira’ and ‘Pinot Noir’, the degree of susceptibility to *Bc* is significantly associated to CW-related mechanisms specific to the berry stage and the cultivar. In fact, ‘Trincadeira’ already showed visible symptoms at the green berry stage (E-L 33), correlating with a significant induction of *VvPG22* (Agudelo-Romero et al. 2015). This result is consistent with the identification of two specific members of *PG* and *EXP* gene families, which have been implicated in the susceptibility of tomato fruit to *Bc* (Cantu et al. 2009). Moreover, ‘Pinot Noir’, when infected with *Bc* at the flowering stage (EL25/EL26), induces within 96 hours the expression of genes encoding germin-like proteins and proline-rich extensin-like proteins, involved in CW toughening, leading *Bc* into quiescence (Haile et al. 2017). In the second phase, in the mature ‘Pinot Noir’ berry, the pathogen emerges and increases the induction of *PME* and *PG* genes, involved in the physiological CW loosening during ripening, favoring its spread (Haile et al. 2020).

**Fig. 3:**
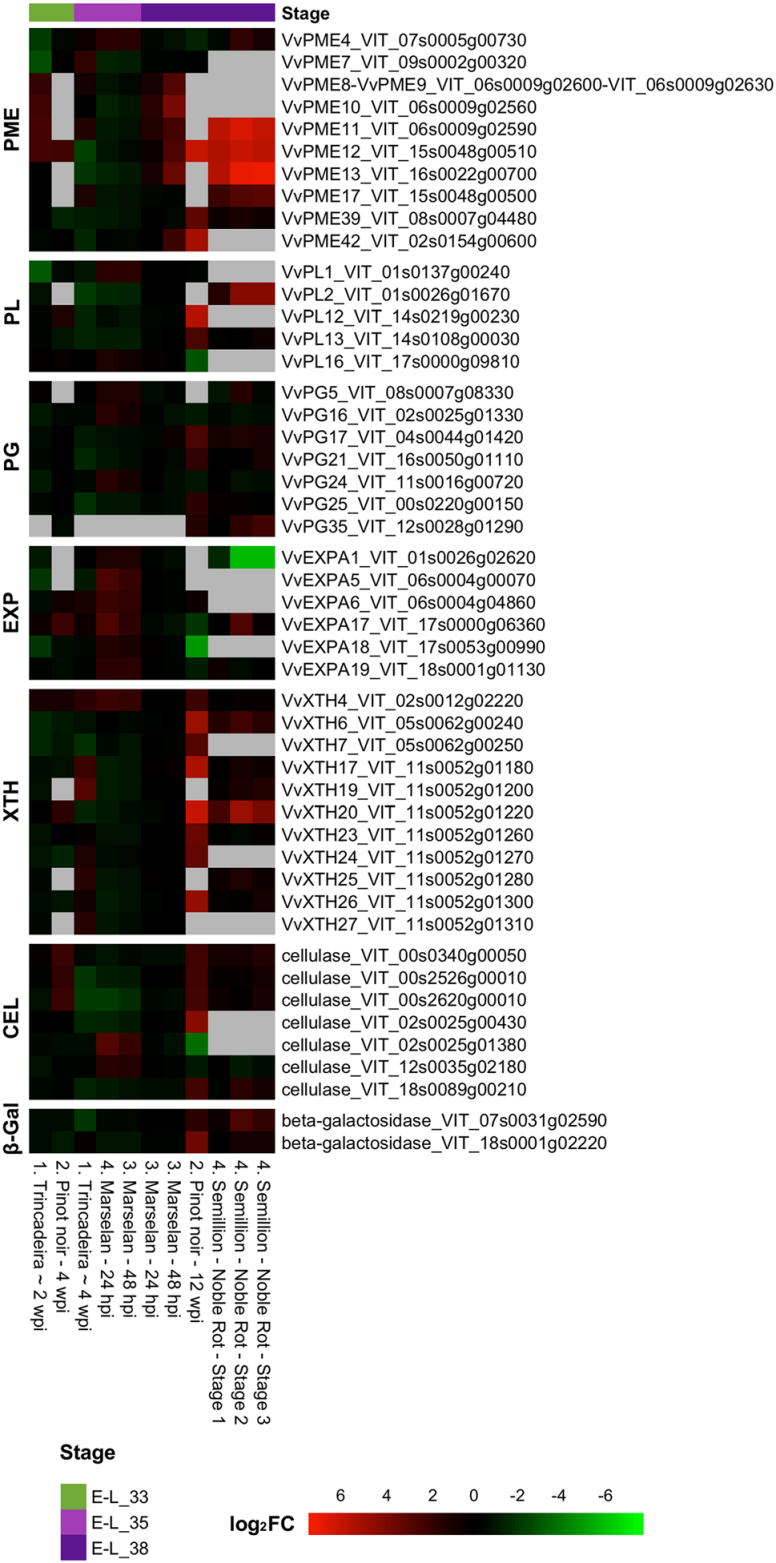
Expression heatmap of the eight most important classes of grapevine CWME-encoding genes showing the most interesting profile in 12 contrasts of the grapevine gene expression compendium VESPUCCI (Moretto et al. 2022). The heatmap visualizes the most significant results of those obtained and visualized in Online Resource 2. Each column of the heatmap corresponds to a contrast comparing B. cinerea infected vs. control samples. The accession numbers of the experiments retrieved by GEO (http://www.ncbi.nlm.nih.gov/geo/) and SRA (https://www.ncbi.nlm.nih.gov/sra) are the following: (1) GSE52586; (2) PRJNA414966; (3) GSE65969; (4) PRJNA281236. The color scale indicates the log2 expression ratio of each test (infected sample) vs. the reference condition (control sample) within each experiment. The genes are considered significantly differentially expressed if they fulfill a p-value of < 0.01 and an absolute log2 Fold Change (FC) value ≥ 1.0). E–L = Modified E–L (Eichhorn and Lorenz) system for grapevine growth stages from (Coombe 1995 ). For the abbreviations of the CWMEs refer to Table 1 . The heatmap was created using RStudio (ver 2023.09.1 + 494, R ver 4.3.2)

Based on these previous studies, we propose here a comparative expression analysis of the eight most important classes of grapevine CWME-encoding genes during the interaction between the berry and *Bc* (gene list given in Online Resource 1), taking advantage of the publicly available experiments stored in the VESPUCCI compendium (Moretto et al. 2022). All the results obtained are visualized in Online Resource 2, while the most significant results are presented in the heatmap of Fig. 3. The transcriptomic data correspond to four published experiments on grapes at three different berry stages (E-L 33, 35, and 38) and at different times after artificial *Bc* infection, from different susceptible grapevine cultivars (‘Marselan’, ‘Trincadeira’, ‘Semillon’ and ‘Pinot Noir’).

Specific CWME-encoding genes, from most of the eight classes, show a significantly higher expression in ripe berries, both in the case of bunch and noble rot development (last four columns of the heatmap). Noble rot results from atypical *Bc* infections of ripe or overripe grape berries, under specific environmental conditions, and promotes the accumulation of aroma and flavor compounds, which are essential for the production of high-quality dessert wines known as botrytized wines (Blanco-Ulate et al. 2015; Lovato et al. 2019).

The analysis of the expression profile in ‘Pinot Noir’ ripe berries at 12 weeks post-inoculation (wpi) showing *Bc* symptoms, or in ‘Semillon’ berries during noble rot development, compared to that in ‘Marselan’ berries at 48 h post-inoculation (hpi), shows that the presence of the fungus for long periods induces a higher number of *CWME* genes with significantly higher intensity compared to control conditions. This is the case for *VvPME10* (Haile et al. 2020; Blanco-Ulate et al. 2015), *VvPME11, VvPME12, VvPME13, VvPME39 and VvPME42*; *VvPL2*, *VvPL12* and *VvPL13; VvPG17, VvPG21, VvPG25*, and *VvPG35; VvXTH6, VvXTH7, VvXTH17, VvXTH20, VvXTH23, VvXTH24*, and *VvXTH26*; five cellulase members (VIT_00s0340g00050, VIT_00s2526g00010, VIT_00s2620g00010, VIT_02s0025g00430, VIT_18s0089g00210*)* and two β-galactosidases *(*VIT_07s0031g02590 and VIT_18s0001g02220). Regarding the *PME* members, *VvPME12* and *VvPME42* seem to be induced with higher intensity in ‘Pinot Noir’ berries, and in ‘Semillon’ berries the expression of another subset of genes (*VvPME11*, *VvPME12*, and *VvPME13*) is even stronger, although the two cultivars have a similar susceptibility. In ripe berries, shorter times from the inoculation (a few hours or a few weeks) appear to activate what could be a rapid initial host response. In this view, the activation of these genes could be a direct response to the presence of the fungus. With this in mind, some *PME* gene members (*VvPME8, VvPME9*, *VvPME10*) showed a mild induction of expression by the presence of the fungus, suggesting their involvement in the early or intermediate stages of the infection. Overall, most of the *PME* gene members identified here as highly induced in ripe and overripe berries upon Bc infection are also highly expressed under normal conditions (Fasoli et al. 2012), suggesting that their increased expression during berry softening could make berries more susceptible.

However, the significant induction of the previously mentioned gene members, both at longer and shorter times, is not a consistent behavior in all CWDE gene families. In fact, it’s evident that genes belonging to the *EXP* gene family, show an increased expression during the initial and intermediate stage of infection (*VvEXPA1*, *VvEXPA5*, *VvEXPA6*, *VvEXPA17*, *VvEXPA18*, *VvEXPA19*), as seen in the infected ‘Marselan’ berries at véraison. Interestingly, the expression of *VvEXPA17*, *VvEXPA18*, *and VvEXPA19* is downregulated at maturity in ‘Pinot Noir’, and *VvEXPA1* continues to decrease as the infection progresses (Fig. 3, Semillon - Noble Rot stages). This *EXP* gene expression behavior may be related to the specific host developmental stages or the specific infection stages, as previously reported (Fasoli et al. 2016; Haile et al. 2020). Indeed, this evidence is consistent with that indicating that *Bc* undergoes an initial colonization step, followed by a quiescent phase during the green berry stages, until the fungal egression at maturity (Haile et al. 2020).

**Fig. 4:**
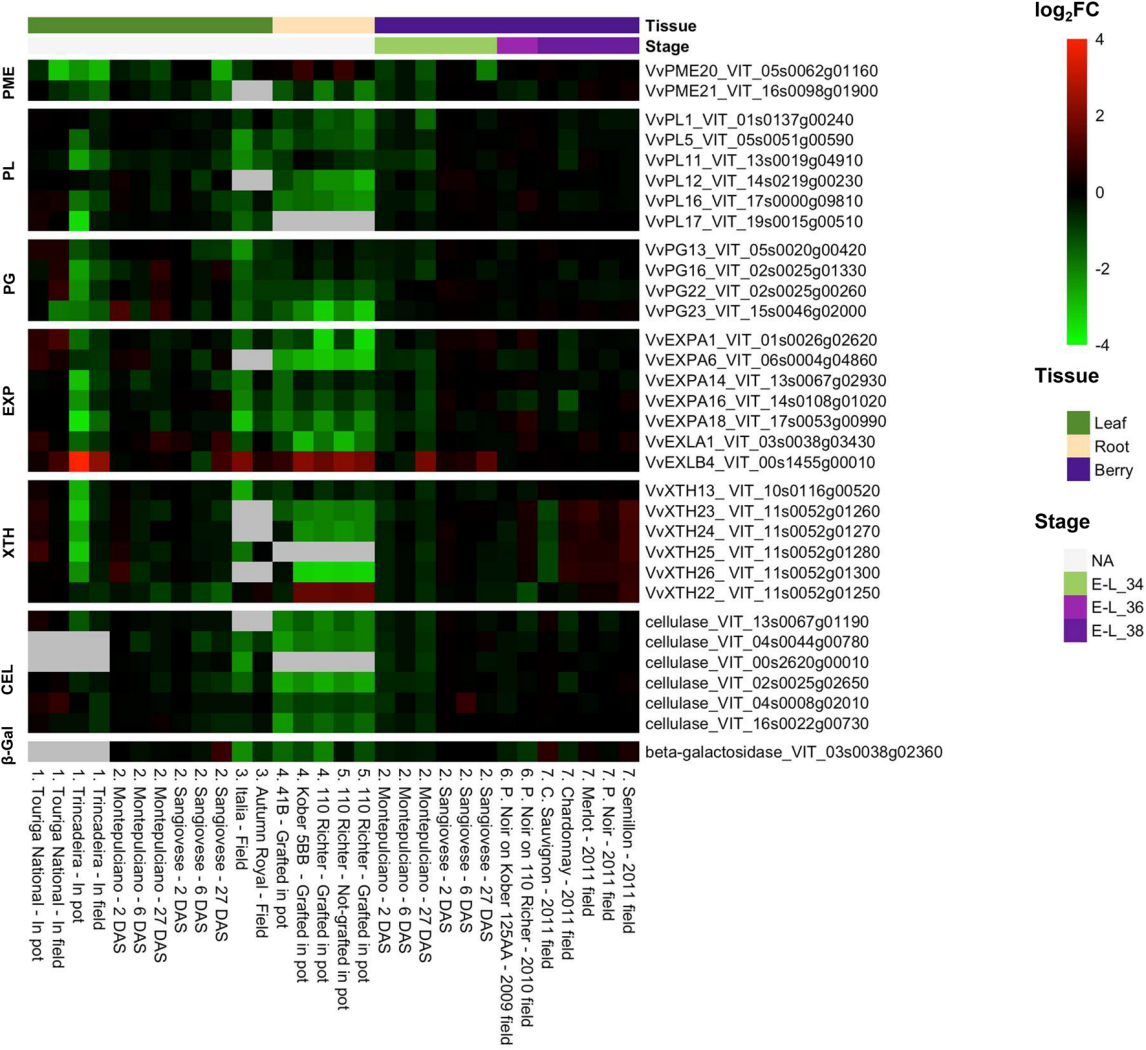
Expression heatmap of the eight most important classes of grapevine CWME-encoding genes showing the most interesting profile in 30 contrasts of the grapevine gene expression compendium VESPUCCI (Moretto et al. 2022). The heatmap visualizes the most significant results of those obtained and visualized in Online Resource 3. Each column of the heatmap corresponds to a contrast comparing water-stressed vs. non-water-stressed conditions in leaf, root, and berry. The accession numbers of the experiments retrieved by GEO (https://www.ncbi.nlm.nih.gov/geo/) and SRA (https://www.ncbi.nlm.nih.gov/sra) are the following: (1) GSE57669; (2) GSE70670; (3) GSE126052; (4) GSE89075; (5) GSE89185; (6) GSE66391; (7) GSE72421. The color scale indicates the log2 expression ratio of each test (water-stressed sample) vs. the reference condition (control sample) within each experiment. The genes are considered significantly differentially expressed if they fulfill ap-value of < 0.01 and an absolute log2 Fold Change (FC) value ≥ 1.0). Water limitation conditions differ among the selected experiments. DAS = days after the beginning of stress application. For the abbreviation of the eight gene families, refer to Table 1 . The heatmap was created using RStudio (ver 2023.09.1 + 494, R ver 4.3.2)

In conclusion, many members of CWDE-encoding gene families, especially *PME, PL* and *PG* genes, show a certain modulation upon *Bc* infection, confirming their putative role in grapevine berry-*Bc* interaction, as shown by several works in the literature (Bethke et al. 2014; Lionetti et al. 2015, Corpo et al. 2020), and highlighted in other fleshy fruit crops such as tomato (Cantu et al. 2008a; Ortega-Salazar et al. 2023) and strawberry (Zhang et al. 2022; López-Casado et al. 2023). However, future functional studies are needed to prove the role of these specific genes in the grape berry response to *Bc*.

### CW modifications in response to water stress

CW remodeling is one of the several mechanisms activated by plants under water stress (Le Gall et al. 2015). A general response to minimize water loss is the thickening of the CW, at the expense of CW extensibility, which negatively affects cell and tissue growth (Jogawat et al. 2021). It’s interesting to note that the plants that are best adapted to cope with drought conditions can grow at reduced water potential, and at least for some organs or tissues, retain the ability to relax and extend the cell wall at low turgor pressure (Moore et al. 2008).

In grapevine, the response to drought is a multifactorial trait, involving manifold metabolic pathways. Moreover, it is genotype dependent, with some genotypes orchestrating a very early and broad transcriptomic response to water deficit, implying the modulation of several gene ontology categories, while others show a limited and late response, involving the modulation of a tiny set of genes (Rocheta et al. 2016; Catacchio et al. 2019; Carvalho et al. 2023; Bianchi et al. 2023; Hewitt et al. 2023). Therefore, since grapevine is cultivated in the form of a grafted plant, both the cultivar used as scion, the rootstock, and their combination are key factors to consider, as shown by many studies evaluating the physiological and transcriptomic effects of applying water limitation regimes to *Vitis spp.* genotypes (Ghan et al. 2015; Berdeja et al. 2015; Dal Santo et al. 2016; Rocheta et al. 2016; Haider et al. 2017; Carvalho et al. 2022, 2023; Yıldırım et al. 2018; Catacchio et al. 2019; Cochetel et al. 2020; Bianchi et al. 2023; Hewitt et al. 2023).

Furthermore, organ-specific gene expression profiles emerged from the same studies, indicating the modulation of specific regulatory networks in leaves, berries, and roots, responsible for different physiological effects. In leaves, in addition to the CW organization, the main activated networks concern (i) ABA biosynthesis and signaling leading to stomatal closure, (ii) soluble sugar synthesis and mobilization to maintain osmotic balance and cell turgor, (iii) reactive oxygen species (ROS) scavenging enzymes to counteract oxidative stress (Dal Santo et al. 2016; Rocheta et al. 2016; Catacchio et al. 2019; Carvalho et al. 2023). In berries, some of the main categories modulated by water deficit are (i) cell wall, (ii) hormones, and (iii) secondary metabolites, while, in the root compartment, the regulatory networks affected by drought include (i) CWMEs, which were generally repressed (Yıldırım et al. 2018), (ii) sugar and protein transporters, whose induction may allow carbohydrate and nitrogen accumulation (Yıldırım et al. 2018; Corso et al. 2015), and (iii) osmolyte producers whose up-regulation could facilitate root osmotic adjustment (Yıldırım et al. 2018).

As done in the previous paragraph related to CW modifications upon *Bc* infection, we explored the expression profiles of the eight most important classes of CWME enzymes (Online Resource 1) upon water stress conditions. Transcriptomic data, collected from different organs at different developmental stages, were retrieved from published studies conducting water stress experiments in *V. vinifera* cultivars and in rootstocks and visualized in the heatmaps in Fig. 4 and Online Resource 3.

**Fig. 5:**
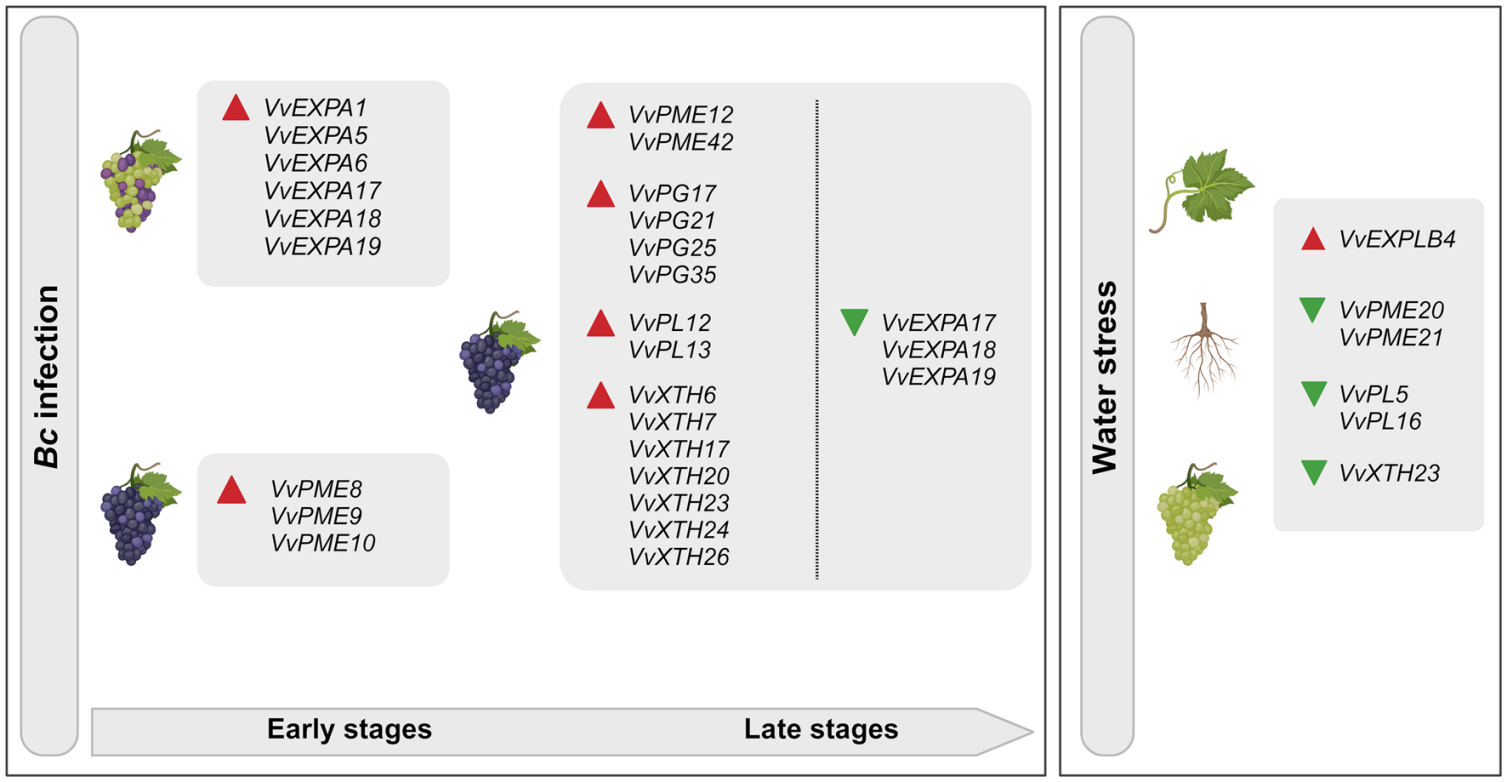
Schematic representation of the main CWME-encoding genes putatively involved in the berry-compatible interaction with *Bc*, both at early and late stages of infection, and under water stress conditions. The upper part of the figure focuses on the gene members most modulated during Bc infection in the susceptible wine cultivars ‘Marselan’ (at 48 hpi) (Kelloniemi et al. 2015) and ‘Pinot noir’ (at 12 wpi) (Haile et al. 2020). The lower part of the figure indicates the genes modulated under water stress conditions and shared by different organs: leaves (Catacchio et al. 2019), berries (Dal Santo et al. 2016) and roots (Yıldırım et al. 2018). Red triangles: up-regulation; green triangles: down-regulation. The artwork was created using BioRender.com

CWME-encoding genes are generally down-regulated during water stress, especially in leaves and roots, possibly leading to a stiffer cell wall matrix with highly methyl-esterified pectin, resulting, at the whole plant level, in a reduction of shoot and root growth as previously reported by Moore et al. (2008) and Haas et al. (2020). Although our comparative analysis in roots includes only five experiments, in which the roots of three commonly used rootstock genotypes are analyzed, the observed gene modulation is quite striking. A strong down-regulation of almost all genes considered is evident, suggesting that water stress is perceived to a greater extent in the underground organs of the plant, where it leads to profound CW remodeling. However, specific members of the *EXP* and *XTH* families (namely *VvEXLB4* and *VvXTH22*) resulted highly over-expressed in roots of the rootstock 110 Richter and Kober 5BB, subjected to water stress (Yıldırım et al. 2018). Such enzymes, which are key regulators of CW expansion, may promote a certain degree of CW loosening needed for maintaining cell extensibility and expansion during root elongation in drought-tolerant genotypes under stress (Yıldırım et al. 2018). Interestingly, *VvEXLB4* is also induced in leaves of water-stressed ‘Trincadeira’ (Rocheta et al. 2016) and ‘Italia’ (Catacchio et al. 2019), two varieties with a conservative behavior under drought, which activate a strong and fast transcriptomic modulation in response to stress. Moreover, *VvEXLB4* and *VvEXLB2* are also induced in the berries of both ‘Montepulciano’ and ‘Sangiovese’, maintained at 40% of water availability for 27 days (Dal Santo et al. 2016). A role in promoting stress tolerance by some *EXP* and *XTH* members has also been reported in the literature for several other crops, such as rice and wheat (Cho and Kende 1997; Choi et al. 2006; Yang et al. 2006).

Considering each class of CWMEs, we highlighted the most significantly modulated genes with a similar profile in different experiments (Fig. 4). Regarding PME, *VvPME20* and *VvPME21* are the most down-regulated genes in leaves and roots, respectively. Regarding *PL* genes, *VvPL5*, *VvPL11*, and *VvPL17* are strongly down-regulated in leaves of ‘Trincadeira’ and ‘Italia’ while in roots of ‘110 Richter’, the most down-regulated genes are *VvPL4*, *VvPL5*, *VvPL13, VvPL16*. Regarding PG, *VvPG13, VvPG16*, *VvPG22*, and *VvPG23* are repressed in leaves of ‘Trincadeira’ and ‘Italia’, while in roots only *VvPG23* shows a significant down-regulation. The most repressed *EXPs* in leaves are *VvEXPA14*, *VvEXPA16*, and *VvEXPA18*, while in roots they are *VvEXPA1*, *VvEXPA6*, *VvEXPA18*, and *VvEXLA1*. Regarding *XTHs*, the most down-regulated members are *VvXTH13*, *VvXTH23*, and *VvXTH25*, while in roots are *VvXTH23*, *VvXTH24*, and *VvXTH26*. Strong repression is also observed for some cellulase and β-galactosidase members, especially in roots.

It is also worth mentioning the role of the CW in guard cells, which delimit stomatal pores that undergo repeated cycles of swelling and deflation in response to changes in turgor pressure. Stomata control plant transpiration and gas exchange with the atmosphere, and their regulation is essential for plants to cope with abiotic stress (Hetherington and Woodward 2003). Stomatal opening/closing dynamics are controlled by the hormone ABA and are highly variable among grapevine genotypes (Schultz 2003; Faralli et al. 2022). A tighter control of the stomatal aperture is characteristic of a near-isohydric behavior, as in ‘Montepulciano’, ‘Trincadeira’, ‘Italia’ while a delayed regulation is typical of anisohydric phenotypes, as in the case of ‘Sangiovese’. One factor that may influence the timing of stomatal regulation is the composition and architecture of the CWs in the guard cells. A CW with a high degree of methylesterification is stiffer, and, consequently, the mechanical adjustments of guard cells shape would be slower, as shown in *pme6 Arabidopsis* mutants where the guard cells with a CW enriched in methylesterified pectin prevented the rapid adjustment of cell shape (Amsbury et al. 2016). On the contrary, a CW with de-esterified pectin, with shorter homogalacturonan chains may fasten stomatal closure and opening (Yi et al. 2018).

### Examples of biotechnological manipulation of the CW properties in fruit trees

The important role of CWs in fruit ripening and quality determination has generated considerable interest in targeting this cellular structure for genetic modification. Rapid progress in the discovery and characterization of the major CWMEs, initially in a model species such as *A. thaliana*, but more recently also in several crops with fleshy fruits, has led many groups to use them as targets for biotechnological applications. These studies have exploited the down-regulation/knock-out of specific members of the main CWME-encoding gene families by antisense, RNAi, or CRISPR/Cas9 approaches, or their overexpression under a strong constitutive promoter, to analyze their role in fruit quality, as well as in the response to biotic stresses. The fruits of the genetically modified plants were in most cases tested for softening/firmness traits and related CW biochemical processes (e.g. pectin and hemicellulose depolymerization, pectin solubilization), but, in some cases, the phenotyping also included resistance to pathogens, mainly *B. cinerea*. Table 2 summarizes the principal results of such studies, which were mainly conducted in tomato and strawberry, and to a lesser extent also in perennial fruit trees such as peach, apple, and grapevine.

**Table 2:** List of the most important studies related to the characterization of the role of CW in fruit softening and susceptibility to *B. cinerea* in several crops with fleshy fruit

Fruit crop	Gene family	Gene	Strategy	Fruit softening	Susceptibility to Bc	Other Effects	References	Putative grapevine orthologue
Tomato	Pectate lyase	*SIPL *Solyc03g111690	RNAi	Reduced/delayed	Reduced		Uluisik et al. (2016), Yang et al. (2017)	*VvPL16 *VIT_17s0000g09810
			CRISPR/Cas9	Reduced/delayed	NA		Wang et al. (2019)	
		*SIPL16 *Solyc06g083580	RNAi	Reduced/delayed	NA	Loss of weight	Ren et al. (2023)	*VvPL6 *VIT_07s0005g05520
	Rhamnogalacturonan lyase	*SlRGL *SolyC11g011300	OE	Reduced/delayed	NA	lower number of seeds and fruits, higher root length, less pollen germination and viability	Ochoa-Jiménez et al. (2018)	VIT_00s0346g00030
	Polygalacturonase	*SlPGa2 *Solyc10g080210	CRISPR/Cas9	Unaffected	NA	Decreased color index, higher fruit weight	Wang et al. (2019)	*VvPG5 *VIT_08s0007g08330
		*SIPG *Solyc10g080210	CRISPR/Cas9	Reduced/delayed	NA		Nie et al. (2022)	*VvPG5 *VIT_08s0007g08330
	Pectate lyase and Polygalacturonase	*SlPG2a *Solyc10g080210 *SlPL *Solyc03g111690	CRISPR double mutants	Reduced/delayed (additive effect of the two genes)	Reduced	Improved fruit quality traits	Ortega-Salazar et al. (2023)	*VvPG5 *VIT_08s0007g08330 + *VvPL16 *VIT_17s0000g09810
	Polygalacturonase and Expansin	*LePG LeExp1*	antisense and RNAi	Reduced/delayed	Reduced		Powell et al. (2003), Cantu et al. (2008a)	Nd
	Xyloglucan- endotransglucosylase/hydrolase	*SlXTH5 *Solyc01g081060	CRISPR/Cas9	Slightly reduced		decreased color index	Wang et al. (2023)	*VvXTH4 *VIT_02s0012g02220
		*SlXTH1*	OE	Reduced			Miedes et al. (2010)	Nd
	β-galactosidase	*SlTBG4 *Solyc12g008840	CRISPR/Cas9	Unaffected			Wang et al. (2019)	VIT_11s0016g02200
Strawberry	Pectate lyase	*FvePL1 *FvH4_2g19540 *FvePL4 *FvH4_4g05760 *FvePL7 *FvH4_5g06720	RNAi	Reduced/delayed		Reduced leaf size, altered petal architecture, partial male sterility	Huang et al. (2023)	*VvPL6 *VIT_07s0005g05520 *VvPL5 *VIT_05s0051g00590 *VvPL16 *VIT_17s0000g09810
		*FaPLc*	Antisense	Reduced			Jiménez-Bermúdez et al.( 2002), Posé et al. (2015)	Nd
		*FvPLA *FvH4_4g05760	RNAi	Reduced			Zhang et al. (2022)	*VvPL5 *VIT_05s0051g00590
	Pectate lyase and endoglucase	FaPLC + FaEG3	Antisense	Reduced		Reduced yield, reduced fruit weight	Youssef et al. (2013)	Nd
	Pectin methylesterase	FvPME38 MK775554 FvPME39 MK775555	RNAi	Reduced/delayed			Xue et al. (2020)	*VvPME29 *VIT_09s0002g00330 *VvPME7 *VIT_09s0002g00320
			OE	Enhanced				
	β-galactosidase	FaβGal4 KR189030	Antisense	Partially reduced			Paniagua et al. (2017)	VIT_18s0001g02220
	Polygalacturonase	*FaPG1 *AF380299	Antisense and CRISPR/Cas9	Reduced		Fruit with reduced transpiration	Posé et al. (2013), López-Casado et al. (2023)	*VvPG33 *VIT_12s0057g00320
	Xyloglucan-endotransglucosylase/hydrolase	*FvXTH9 *XP_004293486 *FvXTH6 *XP_004288290	OE via agroinfiltration	Enhanced/accellerated			Witasari et al. (2019)	*VvXTH31 *VIT_12s0134g00160 *VvXTH3 *VIT_01s0150g00460
Peach	Pectate lyase	*PpePL1 *Prupe.1G060900 *PpePL15 *Prupe.5G161300	RNAi (VIGS)	Reduced/delayed			Xu et al. (2022)	*VvPL5 *VIT_05s0051g00590 *VvPL16 *VIT_17s0000g09810
Apple	Polygalacturonase	*MdPG1*	Antisense	Reduced		Reduced water loss	Atkinson et al. ( 2012)	Nd
		*MdPG1 *MD10G1179100	OE	Accellerated		Extensive water loss	Gunaseelan et al. (2023)	Nd
	Expansin	*MdEXLB1*	OE	Accellerated		Reduced plant height	Chen et al. (2022)	Nd
Grapevine	Pectate lyase	*VvPL11 *VIT_213s0019g04910	OE in tomato	Enhanced			Li et al. (2023)	
		*VvPL16 *VIT_217s0000g09810	OE in tomato	Enhanced/accellerated			Ma et al. (2023)	
		*VvPL5 *VIT_205s0051g00590	OE in tomato	Enhanced			Yu et al. (2023)	

The putative grapevine orthologue to the specific CWME-encoding gene has been identified by homology search using the BLAST tool against the 12X.v1 structural annotation of the 12X.0 grapevine genome assembly (https://grapedia.org/genomes/).

It appears that fruit firmness is controlled by a small number of CWMEs and that the impairment of PL and PG activities, consistently across different studies and species, results in firmer fruit, in some cases with a reduced susceptibility to *Bc* and a limited water loss. In particular, silencing of *SlPL16, SlPL*, and *SlPG2a* genes (whose putative orthologs in grapevine are *VvPL6*, *VvPL16*, and *VvPG5*, respectively), alone or in combination, by RNAi or gene editing approaches, significantly reduces fruit softening and susceptibility to *Bc* in tomato (Uluisik et al. 2016; Yang et al. 2017; Wang et al. 2019; Ren et al. 2023; Nie et al. 2022, (Ortega-Salazar et al. 2023). Similarly, tomato fruits, with reduced *LePG* and *LeExp1* expression, were significantly firmer throughout ripening, were less susceptible to deterioration during long-term storage (Powell et al. 2003; Cantu et al. 2008a), and less susceptible to *B. cinerea* attack (Cantu et al. 2008b). On the contrary, XTH and β-galactosidases seem to play a minor role in inducing fruit softening (Wang et al. 2019, 2023), and, in some cases, a higher activity of the enzymes correlates with higher firmness (Miedes et al. 2010). In strawberry, in addition to specific *PLs* and *PGs* (Huang et al. 2023; Jiménez-Bermúdez et al. 2002; Posé et al. 2015; Zhang et al. 2022), *PME* genes were also found to be involved in the process of pectin degradation and fruit softening (Xue et al. 2020).

Regarding grapevine, only a few functional genomics studies have been published recently, all focused on the *PL* gene family and based on the overexpression of *PL* gene members in heterologous systems. Li et al. (2023), by overexpressing *VvPL11*^2^ (VIT_13s0019g04910) in tomato plants, obtained transgenic tomato fruits that were softer than wild type (WT). The same year, Ma and colleagues overexpressed *VvPL16* (VIT_17s0000g09810) in Arabidopsis plants. They found that water- and acid-soluble pectin were significantly higher in the leaves of transgenic plants compared to WT, demonstrating that this gene may promote pectin degradation. In addition, by overexpressing the same gene in tomato, they observed an accelerated fruit softening, suggesting a role for *VvPL16* in inducing CW degradation and fleshy fruit softening. In another study, Yu et al. (2023) overexpressed *VvPL5* (VIT_05s0051g00590) in tomato and obtained fruits with a softer flesh compared to WT at the “orange” and “red” ripening stages. According to publicly available transcriptomic data, *VvPL5* (VIT_05s0051g00590) and *VvPL16* (VIT_17s0000g09810) are the most expressed *PLs* during berry softening in both wine varieties, such as ‘Pinot Noir’ and ‘Cabernet Sauvignon’ (Fasoli et al. 2018), and table grape varieties, such as ‘Thompson seedless’ (Balic et al. 2018) and ‘Muscat Hamburg’ (Ma et al. 2020).

It’s worth noting that the putative grapevine orthologous genes to those that play a key role in inducing fruit softening in other fruit crops (Table 2) are *VvPL1*6, *VvPL6, and VvPL5*. These genes, especially *VvPL5* and *VvPL16*, were significantly down-regulated in the leaves of water-stressed ‘Trincadeira’ and ‘Italia’ and in the roots of water-deprived rootstocks (Figs. 4 and 5). In addition, *VvPL1*6 also seems to be modulated during *Bc* infection (Fig. 3). Therefore, *VvPL* specific genes may be interesting targets for gene editing approaches to improve qualitative and agronomic traits in grapevine. It might be speculated that their specific knock-out would result in plants with firmer berries that can also better tolerate conditions of water shortage and biotic pressure. Such traits can be very attractive for the table grape market, as firmness and the resulting longer shelf life are desired traits by consumers. Conversely, for the wine industry, firmer berries may hinder the extractability of secondary metabolites. Indeed, during wine production, breaking down the CW of berries is necessary to allow the release of metabolites - mainly sugars and acids- from the pulp cells, and polymers-such as pigments, pectin, proteins, and polyphenols-mainly from the skin cells, into the must (Gao et al. 2019). CWMEs from fungal sources are often added, to aid in the CW deconstruction and the extraction of critical polyphenols (tannins and anthocyanins) (Ducasse et al. 2010). Among the most effective enzymes are rhamnogalacturonan lyase and PLs, which are involved in the specific degradation of pectin (Gao et al. 2016). Tannins have a high affinity for pectin (Renard et al. 2017) and this interaction strongly influences their extractability, but also their bioavailability in the final wine, since the phenolic compounds, bound to the pectic fraction, may precipitate during the vinification stages. In this perspective, increasing the ability to manipulate pectin content and degradation, and fine-tuning the processes of polyphenol extraction and precipitation in red wines, are crucial goals of the oenological research (Osete-Alcaraz et al. 2022). Moreover, the de-methylesterification of pectin releases high levels of methanol during the storage of grape pomace until distillation, which significantly influences the composition of the final product (Zocca et al. 2007). For this reason, PMEIs could be used to reduce the formation of methanol in must and pomace, as well as in products derived from fermentation and distillation (https://patents.google.com/patent/WO2008104555A1/en).

## Conclusions and future perspectives

In the context of climate change, with an expected general increase in temperatures and the probable emergence of drought conditions and biotic pressure in many wine-growing regions, grapevine productivity and grape quality may be negatively affected (Droulia and Charalampopoulos 2021). Climate change is a major challenge for the viticultural sector, and one of the strategies to mitigate its potential negative effects is to improve specific plant traits through the application of new genomic techniques such as gene editing. As discussed in detail in the review, the CW is a cellular component that is highly regulated under biotic and abiotic stresses. Therefore, the manipulation of specific genes involved in the processes of formation and modification of the CW could be a compelling strategy to increase the tolerance of grapevines to drought and heat stress (Ezquer et al. 2020) and could also beneficially contribute to the control of the disease caused by the fungus *B. cinerea*, whose spread is strongly influenced by changing environmental conditions (Ciliberti et al. 2015). This review, which provides a compendium of the main CWME-encoding gene members modulated during grapevine berry development, and under biotic and abiotic stress conditions (as summarized in Fig. 5), can be considered a useful guide for the design of specific biotechnological interventions aimed at improving grapevine quality traits and adaptation to a changing climate. The design of these interventions should consider not only the adaptation and productivity of the plant but also the oenological and organoleptic characteristics of the berries, which are closely related to CW composition. Such aspects are determinants for the wine and table grape industry and shape the high socio-economic value of the grapevine.

### Supplementary Information

Below is the link to the electronic supplementary material.

Online Resource 1. List of the eight major classes of grapevine CWME-encoding genes. Gene symbols and gene IDs for expansin (EXP), pectin methylesterase (PME), endo-polygalacturonase (PG), pectate lyase (PL), xyloglucan-endotransglucosylase/hydrolase (XTH) gene families were obtained from the references listed in the last column of the table. For the rhamnogalacturonate lyase (RGL), cellulase (CEL), and β-galactosidase (β-GAL) gene families, they were identified using the hmmscan tool from HMMER 3.1 (Finn et al. 2011) and the Pfam-A database (release 35) against the 12X.v1 structural annotation of the 12X.0 grapevine genome assembly (https://grapedia.org/genomes/). The results were filtered for the Pfam domains PF06045, PF00759, and PF01301 for the RGL, CEL, and β-GAL families, respectively, considering a sequence E-value ≤ 1x10-5. Supplementary material 1 (XLSX 22.5 kb)

Online Resource 2. Expression heatmaps of the eight major classes of grapevine CWME-encoding genes in 12 contrasts of the grapevine gene expression compendium VESPUCCI (Moretto et al. 2022). Each column of the heatmap corresponds to a contrast comparing B. cinerea infected vs. control samples. The accession numbers of the experiments retrieved by GEO (http://www.ncbi.nlm.nih.gov/geo/) and SRA (https://www.ncbi.nlm.nih.gov/sra) are as follows: 1. GSE52586; 2. PRJNA414966; 3. GSE65969; 4. PRJNA281236. The color scale indicates the log2 expression ratio of each test (infected sample) vs the reference condition (control sample) within each experiment. Genes are considered differentially expressed if they meet a p-value of< 0.01 and an absolute log2 fold change (FC) value ≥ 1.0). E-L = Modified E-L (Eichhorn and Lorenz) grapevine growth stage system from Coombe, B.G. (1995). A selection of the heatmaps represented here is included in Fig. 3, which highlights the genes showing the most interesting expression profiles in the selected contrasts. The heatmaps were generated using RStudio (ver 2023.09.1+494, R ver 4.3.2). Supplementary material 2 (PDF 591.8 kb)

Online Resource 3. Expression heatmaps of the eight major classes of grapevine CWME-encoding genes in 30 contrasts from the grapevine gene expression compendium VESPUCCI (Moretto et al. 2022). Each column of the heatmap corresponds to a contrast comparing water-stressed vs. non-water-stressed conditions in leaf, root, and berry. The accession numbers of the experiments retrieved from GEO (http://www.ncbi.nlm.nih.gov/geo/) and SRA (https://www.ncbi.nlm.nih.gov/sra) are as follows: 1. GSE57669; 2. GSE70670; 3. GSE126052; 4. GSE89075; 5. GSE89185; 6. GSE66391; 7. GSE72421. The color scale indicates the log2 expression ratio of each test (water-stressed sample) versus. the reference condition (control sample) within each experiment. Genes are considered differentially expressed if they meet a p-value of< 0.01 and an absolute log2 fold change (FC) value ≥ 1.0). Water restriction conditions varied among the selected experiments. DAS= days after start of stress application. A selection of the heatmaps shown here is included in Fig. 4, which highlights the genes showing the most interesting expression profiles in the selected contrasts. The heatmaps were generated using RStudio (ver 2023.09.1+494, R ver 4.3.2). Supplementary material 3 (PDF 655.3 kb)

## Data Availability

Not applicable.

## Footnotes

1 All the grapevine gene IDs in the present review refer to the 12X.v1 structural annotation of the 12X.0 grapevine genome assembly (https://grapedia.org/genomes/).

2 The nomenclature of the grapevine *PL* genes may be different from the one reported in the original papers due to a lack of consensus among different papers. For the ones here adopted, please refer to the Online Resource 1.

## References

[CR1] Agudelo-Romero P, Erban A, Rego C, Carbonell-Bejerano P, Nascimento T, Sousa L, Martínez-Zapater JM, Kopka J, Fortes AM (2015). Transcriptome and metabolome reprogramming in *Vitis vinifera* Cv. trincadeira berries upon infection with *Botrytis*
*cinerea*. J Exp Bot.

[CR2] Alkan N, Fortes AM (2015). Insights into molecular and metabolic events associated with fruit response to post-harvest fungal pathogens. Front Plant Sci.

[CR3] Amsbury S, Hunt L, Elhaddad N, Knox JP, Fleming AJ, Gray JE, Amsbury S, Hunt L, Elhaddad N, Baillie A, Lundgren M, Verhertbruggen Y, Scheller HV, Knox JP, Fleming AJ, Gray JE (2016). Stomatal function requires pectin de-methyl- esterification of the guard cell wall report Stomatal function requires pectin de-methyl-esterification of the guard cell wall. Curr Biol.

[CR4] Anderson CT, Kieber JJ (2020). Dynamic construction, perception, and remodeling of plant cell walls. Annu Rev Plant Biol.

[CR5] André M, Lacampagne S, Barsacq A, Gontier E, Petrel M, Mercier L, Courot D, Gény-Denis L (2021). Physical, anatomical, and biochemical composition of skins cell walls from two grapevine cultivars (*Vitis vinifera*) of Champagne Region related to their susceptibility to *Botrytis Cinerea* during ripening. Horticulturae.

[CR6] Atkinson RG, Sutherland PW, Johnston SL, Gunaseelan K, Hallett IC, Mitra D, Brummell DA, Schröder R, Johnston JW, Schaffer RJ (2012). Down-regulation of POLYGALACTURONASE1 alters firmness, tensile strength and water loss in apple (*Malus* x *Domestica*) fruit. BMC Plant Biol.

[CR7] Atmodjo MA, Hao Z, Mohnen D (2013). Evolving views of pectin biosynthesis. Annu Rev Plant Biol.

[CR8] Baez LA, Tichá T, Hamann T (2022). Cell wall integrity regulation across plant species. Plant Mol Biol.

[CR9] Balic I, Ejsmentewicz T, Sanhueza D, Silva C, Peredo T, Olmedo P, Barros M, Verdonk JC, Paredes R, Meneses C, Prieto H, Orellana A, Defilippi BG, Campos-Vargas R (2014). Biochemical and physiological study of the firmness of table grape berries. Postharvest Biol Technol.

[CR10] Balic I, Vizoso P, Nilo-Poyanco R, Sanhueza D, Olmedo P, Sepúlveda P, Arriagada C, Defilippi BG, Meneses C, Campos-Vargas R (2018). Transcriptome analysis during ripening of table grape berry cv. Thompson Seedless PLoS One.

[CR11] Bellincampi D, Cervone F, Lionetti V (2014). Plant cell wall dynamics and wall-related susceptibility in plant-pathogen interactions. Front Plant Sci.

[CR12] Berdeja M, Nicolas P, Kappel C, Dai ZW, Hilbert G, Peccoux A, Lafontaine M, Ollat N, Gomès E, Delrot S (2015). Water limitation and rootstock genotype interact to alter grape berry metabolism through transcriptome reprogramming. Hortic Res.

[CR13] Bethke G, Grundman RE, Sreekanta S, Truman W, Katagiri F, Glazebrook J (2014). Arabidopsis PECTIN METHYLESTERASEs contribute to immunity against *Pseudomonas*
*syringae*. Plant Physiol.

[CR14] Bianchi D, Ricciardi V, Pozzoli C, Grossi D, Caramanico L, Pindo M, Stefani E, Cestaro A, Brancadoro L, De Lorenzis G (2023). Physiological and transcriptomic evaluation of drought effect on own-rooted and grafted grapevine rootstock (1103P and 101-14MGt). Plants.

[CR15] Blanco-Ulate B, Morales-Cruz A, Amrine KCH, Labavitch JM, Powell ALT, Cantu D (2014). Genome-wide transcriptional profiling of *Botrytis cinerea* genes targeting plant cell walls during infections of different hosts. Front Plant Sci.

[CR16] Blanco-Ulate B, Amrine KCH, Collins TS, Rivero RM, Vicente AR, Morales-Cruz A, Doyle CL, Ye Z, Allen G, Heymann H, Ebeler SE, Cantu D (2015). Developmental and metabolic plasticity of white-skinned grape berries in response to *Botrytis Cinerea* during noble rot. Plant Physiol.

[CR18] Bonawitz ND, Chapple C (2010). The genetics of lignin biosynthesis: connecting genotype to phenotype. Annu Rev Genet.

[CR19] Brummell DA (2006). Cell wall disassembly in ripening fruit. Funct Plant Biol.

[CR20] Brummell DA, Harpster MH (2001). Cell wall metabolism in fruit softening and quality and its manipulation in transgenic plants. Plant Mol Biol.

[CR22] Caffall KH, Mohnen D (2009). The structure, function, and biosynthesis of plant cell wall pectic polysaccharides. Carbohydr Res.

[CR24] Cantu D, Vicente AR, Greve LC, Dewey FM, Bennett AB, Labavitch JM, Powell ALT (2008). The intersection between cell wall disassembly, ripening, and fruit susceptibility to *Botrytis Cinerea*. Proc Natl Acad Sci U S A.

[CR25] Cantu D, Vicente AR, Labavitch JM, Bennett AB, Powell ALT (2008). Strangers in the matrix: plant cell walls and pathogen susceptibility. Trends Plant Sci.

[CR26] Cantu D, Blanco-Ulate B, Yang L, Labavitch JM, Bennett AB, Powell ALT, De Biología E (2009). Ripening-regulated susceptibility of tomato fruit to *Botrytis Cinerea* requires NOR but Not RIN or ethylene. Plant Physiol.

[CR27] Carpita NC, Gibeaut DM (1993). Structural models of primary cell walls in flowering plants: consistency of molecular structure with the physical properties of the walls during growth. Plant J.

[CR28] Carvalho LC, Ramos MJN, Faísca-Silva D, van der Kellen D, Fernandes JC, Egipto R, Lopes CM, Amâncio S (2022). Developmental regulation of transcription in touriga nacional berries under deficit irrigation. Plants.

[CR29] Carvalho LC, Ramos MJN, Faísca-Silva D, Marreiros P, Fernandes JC, Egipto R, Lopes CM, Amâncio S (2023). Modulation of the berry skin transcriptome of cv. tempranillo induced by water stress levels. Plants.

[CR30] Catacchio CR, Alagna F, Perniola R, Bergamini C, Rotunno S, Calabrese FM, Crupi P, Antonacci D, Ventura M, Cardone MF (2019). Transcriptomic and genomic structural variation analyses on grape cultivars reveal new insights into the genotype-dependent responses to water stress. Sci Rep.

[CR31] Chen Y, Xie B, An X, Ma R, Zhao D, Cheng C, Li E, Zhou J, Kang G, Zhang Y (2022). Overexpression of the apple expansin-like gene *MdEXLB1* accelerates the softening of fruit texture in tomato. J Integr Agric.

[CR32] Cho H-T, Kende H (1997). Expansin genes 1 s correlated with growth in deepwater rice. Plant Cell.

[CR33] Choi D, Cho H, Lee Y (2006). Expansins: expanding importance in plant growth and development. Physiol Plant.

[CR34] Ciliberti N, Fermaud M, Roudet J, Rossi V (2015). Environmental conditions affect *Botrytis Cinerea* infection of mature grape berries more than the strain or transposon genotype. Phytopathology.

[CR35] Cochetel N, Ghan R, Toups HS, Degu A, Tillett RL, Schlauch KA, Cramer GR (2020). Drought tolerance of the grapevine, *Vitis Champinii* Cv. Ramsey, is associated with higher photosynthesis and greater transcriptomic responsiveness of abscisic acid biosynthesis and signaling. BMC Plant Biol.

[CR36] Conde C, Silva P, Fontes N, Dias ACP, Tavares RM, Sousa M, Agasse A, Delrot S, Gerós H (2007). Biochemical changes throughout grape berry development and fruit and wine quality. Food.

[CR37] Coombe BG (1976). The development of fleshy fruits. Annu Rev Plant Physiol.

[CR38] Coombe BG (1995). Growth stages of the Grapevine: adoption of a system for identifying grapevine growth stages. Aust J Grape Wine Res.

[CR39] Coombe BG, McCarthy MG (2000). Dynamics of grape berry growth and physiology of ripening. Aust J Grape Wine Res.

[CR40] Corso M, Vannozzi A, Maza E, Vitulo N, Meggio F, Pitacco A, Telatin A, D’Angelo M, Feltrin E, Negri AS, Prinsi B, Valle G, Ramina A, Bouzayen M, Bonghi C, Lucchin M (2015). Comprehensive transcript profiling of two grapevine rootstock genotypes contrasting in drought susceptibility links the phenylpropanoid pathway to enhanced tolerance. J Exp Bot.

[CR41] Cosgrove DJ (2016). Plant cell wall extensibility: connecting plant cell growth with cell wall structure, mechanics, and the action of wall-modifying enzymes. J Exp Bot.

[CR42] Cosgrove DJ (2023). Structure and growth of plant cell walls. Nat Rev Mol Cell Biol.

[CR43] Dal Santo S, Vannozzi A, Tornielli GB, Fasoli M, Venturini L, Pezzotti M, Zenoni S (2013). Genome-wide analysis of the expansin gene superfamily reveals grapevine-specific structural and functional characteristics. PLoS ONE.

[CR44] Dal Santo S, Palliotti A, Zenoni S, Tornielli GB, Fasoli M, Paci P, Tombesi S, Frioni T, Silvestroni O, Bellincontro A, d’Onofrio C, Matarese F, Gatti M, Poni S, Pezzotti M (2016). Distinct transcriptome responses to water limitation in isohydric and anisohydric grapevine cultivars. BMC Genomics.

[CR45] Del Corpo D, Fullone MR, Miele R, Lafond M, Pontiggia D, Grisel S, Kieffer-Jaquinod S, Giardina T, Bellincampi D, Lionetti V (2020). *AtPME17* is a functional *Arabidopsis thaliana* pectin methylesterase regulated by its PRO region that triggers PME activity in the resistance to *Botrytis cinerea*. Mol Plant Pathol.

[CR46] Donaldson LA (2001). Lignification and lignin topochemistry—an ultrastructural view. Phytochemistry.

[CR47] Droulia F, Charalampopoulos I (2021). Future climate change impacts on European viticulture: a review on recent scientific advances. Atmos (Basel).

[CR48] Ducasse MA, Canal-Llauberes RM, de Lumley M, Williams P, Souquet JM, Fulcrand H, Doco T, Cheynier V (2010). Effect of macerating enzyme treatment on the polyphenol and polysaccharide composition of red wines. Food Chem.

[CR49] Ejsmentewicz T, Balic I, Sanhueza D, Barria R, Meneses C, Orellana A, Prieto H, Defilippi BG, Campos-Vargas R (2015). Comparative study of two table grape varieties with contrasting texture during cold storage. Molecules.

[CR50] Engelsdorf T, Gigli-Bisceglia N, Veerabagu M, McKenna JF, Vaahtera L, Augstein F, Van der Does D, Zipfel C, Hamann T (2018). The plant cell wall integrity maintenance and immune signaling systems cooperate to control stress responses in *Arabidopsis thaliana*. Sci Signal.

[CR51] Ezquer I, Salameh I, Colombo L, Kalaitzis P (2020). Plant cell walls tackling Climate change: biotechnological strategies to improve crop adaptions and photosynthesis in response to global warming. Plants.

[CR52] Faralli M, Bontempo L, Bianchedi PL, Moser C, Bertamini M, Lawson T, Camin F, Stefanini M, Varotto C (2022). Natural variation in stomatal dynamics drives divergence in heat stress tolerance and contributes to seasonal intrinsic water-use efficiency in *Vitis vinifera* (subsp. *sativa* and *sylvestris*). J Exp Bot.

[CR53] Fasoli M, Dal Santo S, Zenoni S, Battista Tornielli G, Farina L, Zamboni A, Porceddu A, Venturini L, Bicego M, Murino V, Ferrarini A, Delledonne M, Pezzotti M (2012). The grapevine expression atlas reveals a deep transcriptome shift driving the entire plant into a maturation program. Plant Cell.

[CR54] Fasoli M, Dell’Anna R, Dal Santo S, Balestrini R, Sanson A, Pezzotti M, Monti F, Zenoni S (2016). Pectins, hemicelluloses and celluloses show specific dynamics in the internal and external surfaces of grape berry skin during ripening. Plant Cell Physiol.

[CR55] Fasoli M, Richter CL, Zenoni S, Bertini E, Vitulo N, Dal Santo S, Dokoozlian N, Pezzotti M, Tornielli GB (2018). Timing and order of the molecular events marking the onset of berry ripening in grapevine. Plant Physiol.

[CR56] Finn RD, Clements J, Eddy SR (2011) HMMER web server: interactive sequence similarity searching. Nucl Acids Res 39:29–37.10.1093/nar/gkr367 10.1093/nar/gkr367 PMC3125773 21593126

[CR58] Gao Y, Fangel JU, Willats WGT, Vivier MA, Moore JP (2016). Dissecting the polysaccharide-rich grape cell wall matrix using recombinant pectinases during winemaking. Carbohydr Polym.

[CR59] Gao Y, Zietsman AJJ, Vivier MA, Moore JP (2019). Deconstructing wine grape cell walls with enzymes during winemaking: new insights from glycan microarray technology. Molecules.

[CR60] Ghan R, Van Sluyter SC, Hochberg U, Degu A, Hopper DW, Tillet RL, Schlauch KA, Haynes PA, Fait A, Cramer GR (2015). Five omic technologies are concordant in differentiating the biochemical characteristics of the berries of five grapevine (*Vitis vinifera* L.) cultivars. BMC Genomics.

[CR61] Gilbert HJ (2010). The biochemistry and structural biology of plant cell wall deconstruction. Plant Physiol.

[CR62] Giovannoni J (2001). Molecular biology of fruit maturation and ripening. Annu Rev Plant Physiol Plant Mol Biol.

[CR63] Goulao LF, Fernandes JC, Lopes P, Amâncio S (2012). Tackling the cell wall of the grape berry. Biochem Grape Berry.

[CR64] Grimplet J, Deluc LG, Tillett RL, Wheatley MD, Schlauch KA, Cramer GR, Cushman JC (2007). Tissue-specific mRNA expression profiling in grape berry tissues. BMC Genomics.

[CR65] Gunaseelan K, Schröder R, Rebstock R, Ninan AS, Deng C, Khanal BP, Favre L, Tomes S, Dragulescu MA, O’Donoghue EM, Hallett IC, Schaffer RJ, Knoche M, Brummell DA, Atkinson RG (2023). Constitutive expression of apple endo-POLYGALACTURONASE1 in fruit induces early maturation, alters skin structure and accelerates softening. Plant J Epub Ahead.

[CR66] Haas KT, Wightman R, Meyerowitz EM, Peaucelle A (2020). Pectin homogalacturonan nanofilament expansion drives morphogenesis in plant epidermal cells. Science.

[CR67] Haider MS, Zhang C, Kurjogi MM, Pervaiz T, Zheng T, Zhang C, Lide C, Shangguan L, Fang J (2017). Insights into grapevine defense response against drought as revealed by biochemical, physiological and RNA-Seq analysis. Sci Rep.

[CR68] Haile ZM, Pilati S, Sonego P, Malacarne G, Vrhovsek U, Engelen K, Tudzynski P, Zottini M, Baraldi E, Moser C (2017). Molecular analysis of the early interaction between the grapevine flower and *Botrytis cinerea* reveals that prompt activation of specific host pathways leads to fungus quiescence. Plant Cell Environ.

[CR69] Haile ZM, Malacarne G, Pilati S, Sonego P, Moretto M, Masuero D, Vrhovsek U, Engelen K, Baraldi E, Moser C (2020). Dual transcriptome and metabolic analysis of *Vitis vinifera* Cv. pinot noir berry and *Botrytis cinerea* during quiescence and egressed infection. Front Plant Sci.

[CR70] Hardie WJ, O’Brien TP, Jaudzems VG (1996). Morphology, anatomy and development of the pericarp after anthesis in grape, *Vitis*
*vinifera* L.. Aust J Grape Wine Res.

[CR71] Hetherington AM, Woodward FI (2003). The role of stomata in sensing and driving environmental change. Nature.

[CR72] Hewitt S, Hernández-Montes E, Dhingra A, Keller M (2023). Impact of heat stress, water stress, and their combined effects on the metabolism and transcriptome of grape berries. Sci Rep.

[CR73] Huang XM, Huang HB (2001). Early post-veraison growth in grapes: evidence for a two-step mode of berry enlargement. Aust J Grape Wine Res.

[CR74] Huang XM, Huang HB, Wang HC (2005). Cell walls of loosening skin in post-veraison grape berries lose structural polysaccharides and calcium while accumulate structural proteins. Sci Hortic (Amsterdam).

[CR75] Huang X, Sun G, Wu Z, Jiang Y, Li Q, Yi Y, Yan H (2023). Genome-wide identification and expression analyses of the pectate lyase (PL) gene family in Fragaria vesca. BMC Genomics.

[CR76] Jarvis MC, Briggs SPH, Knox JP (2003). Intercellular adhesion and cell separation in plants. Plant Cell Environ.

[CR77] Jay JM (1996). Modern food microbiology.

[CR78] Jiang F, Lopez A, Jeon S, de Freitas ST, Yu Q, Wu Z, Labavitch JM, Tian S, Powell ALT, Mitcham E (2019). Disassembly of the fruit cell wall by the ripening-associated polygalacturonase and expansin influences tomato cracking. Hortic Res.

[CR79] Jiménez-Bermúdez S, Redondo-Nevado J, Muñoz-Blanco J, Caballero JL, López-Aranda JM, Valpuesta V, Pliego-Alfaro F, Quesada MA, Mercado JA (2002). Manipulation of strawberry fruit softening by antisense expression of a pectate lyase gene. Plant Physiol.

[CR80] Jogawat A, Yadav B, Chhaya, Lakra N, Singh AK, Narayan OP (2021). Crosstalk between phytohormones and secondary metabolites in the drought stress tolerance of crop plants: a review. Physiol Plant.

[CR81] Kelloniemi J, Trouvelot S, Héloir MC, Simon A, Dalmais B, Frettinger P, Cimerman A, Fermaud M, Roudet J, Baulande S, Bruel C, Choquer M, Couvelard L, Duthieuw M, Ferrarini A, Flors V, Le Pêcheur P, Loisel E, Morgant G, Poussereau N, Pradier JM, Rascle C, Trdá L, Poinssot B, Viaud M (2015). Analysis of the molecular dialogue between gray mold (*Botrytis Cinerea*) and grapevine (*Vitis vinifera*) reveals a clear shift in defense mechanisms during berry ripening. Mol Plant-Microbe Interact.

[CR82] Kuchi VS, Sharavani CSR (2019). Fruit physiology and postharvest management of strawberry. IntechOpen.

[CR83] L’enfant M, Domon J-M, Rayon C, Desnos T, Ralet M-C, Bonnin E, Pelloux J, Pau-Roblot C (2015). Substrate specificity of plant and fungi pectin methylesterases: identification of novel inhibitors of PMEs. Int J Biol Macromol.

[CR84] Le Gall H, Philippe F, Domon J-M, Gillet O, Rôme Pelloux J, Rayon C (2015). Cell wall metabolism in response to abiotic stress. Plants.

[CR85] Li Z, Fernie AR, Persson S (2016). Transition of primary to secondary cell wall synthesis. Sci Bull.

[CR86] Li J, Wu Z, Zhu Z, Xu L, Wu B, Li J (2022). *Botrytis cinerea* mediated cell wall degradation accelerates spike stalk browning in Munage grape. J Food Biochem.

[CR87] Li W, He C, Wei H, Qian J, Xie J, Li Z, Zheng X, Tan B, Li J, Cheng J, Wang W, Ye X, Feng J (2023). VvPL11 is a key member of the pectin lyase gene family involved in grape softening. Horticulturae.

[CR88] Lionetti V, Raiola A, Mattei B, Bellincampi D (2015). The grapevine *VvPMEI1* gene encodes a novel functional pectin methylesterase inhibitor associated to grape berry development. PLoS ONE.

[CR89] López-Casado G, Sánchez-Raya C, Ric-Varas PD, Paniagua C, Blanco-Portales R, Muñoz-Blanco J, Pose S, Matas AJ, Mercado JA (2023). CRISPR/Cas9 editing of the polygalacturonase FaPG1 gene improves strawberry fruit firmness. Hortic Res.

[CR90] Lovato A, Zenoni S, Tornielli GB, Colombo T, Vandelle E, Polverari A, Polverari A (2019). Plant and fungus transcriptomic data from grapevine berries undergoing artificially-induced noble rot caused by *Botrytis*
*cinerea*. Postharvest Biol Technol.

[CR91] Ma L, Sun L, Guo Y, Lin H, Liu Z, Li K, Guo X (2020). Transcriptome analysis of table grapes (*Vitis vinifera* L.) identified a gene network module associated with berry firmness. PLoS ONE.

[CR92] Ma Y, Wang C, Gao Z, Yao Y, Kang H, Du Y (2023). VvPL15 is the core member of the pectate lyase gene family involved in grape berries ripening and softening. Int J Mol Sci.

[CR93] Mercado JA, Pliego-Alfaro F, Quesada MA (2011). Fruit shelf life and potential for its genetic improvement. Breeding for fruit quality.

[CR95] Miedes E, Herbers K, Sonnewald U, Lorences EP (2010). Overexpression of a cell wall enzyme reduces xyloglucan depolymerization and softening of transgenic tomato fruits. J Agric Food Chem.

[CR96] Moore JP, Farrant JM, Driouich A (2008). A role for pectin-associated arabinans in maintaining the flexibility of the plant cell wall during water deficit stress. Plant Signal Behav.

[CR98] Moore JP, Nguema-Ona E, Fangel JU, Willats WGT, Hugo A, Vivier MA (2014). Profiling the main cell wall polysaccharides of grapevine leaves using high-throughput and fractionation methods. Carbohydr Polym.

[CR99] Moretto M, Sonego P, Pilati S, Matus JT, Costantini L, Malacarne G, Engelen K (2022). A COMPASS for VESPUCCI: a FAIR way to explore the grapevine transcriptomic landscape. Front Plant Sci.

[CR100] Nie H, Shi Y, Geng X, Xing G (2022). CRISRP/Cas9-mediated targeted mutagenesis of tomato polygalacturonase gene (SlPG) delays fruit softening. Front Plant Sci.

[CR102] Nunan KJ, Davies C, Robinson SP, Fincher GB (2001). Expression patterns of cell wall-modifying enzymes during grape berry development. Planta.

[CR103] Ochoa-Jiménez VA, Berumen-Varela G, Burgara-Estrella A, Orozco-Avitia JA, Ojeda-Contreras ÁJ, Trillo-Hernández EA, Rivera-Domínguez M, Troncoso-Rojas R, Báez-Sañudo R, Datsenka T, Handa AK, Tiznado-Hernández ME (2018). Functional analysis of tomato rhamnogalacturonan lyase gene Solyc11g011300 during fruit development and ripening. J Plant Physiol.

[CR104] Ortega-Regules A, Romero-Cascales I, Ros-García JM, López-Roca JM, Gómez-Plaza E (2006). A first approach towards the relationship between grape skin cell-wall composition and anthocyanin extractability. Anal Chim Acta.

[CR105] Ortega-Regules A, Ros-García JM, Bautista-Ortín AB, López-Roca JM, Gómez-Plaza E (2008). Differences in morphology and composition of skin and pulp cell walls from grapes (*Vitis vinifera* L.): technological implications. Eur Food Res Technol.

[CR106] Ortega-Salazar I, Crum D, Sbodio AO, Sugiyama Y, Adaskaveg AW, Seymour GB, Li X, Wang SC, Blanco-Ulate B (2023). Double CRISPR knockout of pectin degrading enzymes improves tomato shelf-life while ensuring fruit quality. Plants People Planet.

[CR107] Osete-Alcaraz A, Gómez-Plaza E, Pérez-Porras P, Bautista-Ortín AB (2022). Revisiting the use of pectinases in enology: a role beyond facilitating phenolic grape extraction. Food Chem.

[CR108] Paniagua C, Santiago-Domenech N, Kirby AR, Patrick Gunning A, Morris VJ, Quesada MA, Matas AJ, Mercado JA (2017). Structural changes in cell wall pectins during strawberry fruit development. Plant Physiol Biochem.

[CR109] Payasi A, Mishra NN, Chaves ALS, Singh R (2009). Biochemistry of fruit softening: an overview. Physiol Mol Biol Plants.

[CR110] Peppi MC, Fidelibus MW, Dokoozlian N (2006). Abscisic acid application timing and concentration affect firmness, pigmentation, and color of Flame Seedless grapes. HortScience.

[CR111] Petrasch S, Silva CJ, Mesquida-Pesci SD, Gallegos K, van den Abeele C, Papin V, Fernandez-Acero FJ, Knapp SJ, Blanco-Ulate B (2019). Infection strategies deployed by *Botrytis Cinerea*, *Fusarium acuminatum*, and *Rhizopus stolonifer* as a function of tomato fruit ripening stage. Front Plant Sci.

[CR112] Posé S, Paniagua C, Cifuentes M, Blanco-Portales R, Quesada MA, Mercado JA (2013). Insights into the effects of polygalacturonase FaPG1 gene silencing on pectin matrix disassembly, enhanced tissue integrity, and firmness in ripe strawberry fruits. J Exp Bot.

[CR113] Posé S, Kirby AR, Paniagua C, Waldron KW, Morris VJ, Quesada MA, Mercado JA (2015). The nanostructural characterization of strawberry pectins in pectate lyase or polygalacturonase silenced fruits elucidates their role in softening. Carbohydr Polym.

[CR114] Posé S, Paniagua C, Matas AJ, Gunning AP, Morris VJ, Quesada MA, Mercado JA (2019). A nanostructural view of the cell wall disassembly process during fruit ripening and postharvest storage by atomic force microscopy. Trends Food Sci Technol.

[CR115] Powell ALT, Kalamaki MS, Kurien PA, Gurrieri S, Bennett AB (2003). Simultaneous transgenic suppression of LePG and LeExp1 influences fruit texture and juice viscosity in a fresh market tomato variety. J Agric Food Chem.

[CR116] Ren H, Zhao Q, Feng Y, Tang P, Wang Y, Jiang J, Hu C, Wang Y, Cui B, Xie X, Li Y, Zhao X, Gu H, Huang J, Zhang Y (2023). Gene-specific silencing of *SlPL16*, a pectate lyase coding gene, extends the shelf life of tomato fruit. Postharvest Biol Technol.

[CR117] Renard CMGC, Watrelot AA, Le Bourvellec C (2017). Interactions between polyphenols and polysaccharides: mechanisms and consequences in food processing and digestion. Trends Food Sci Technol.

[CR118] Rocheta M, Coito JL, Ramos MJN, Carvalho L, Becker JD, Carbonell-Bejerano P, Amâncio S (2016). Transcriptomic comparison between two *Vitis vinifera* L. varieties (Trincadeira and Touriga Nacional) in abiotic stress conditions. BMC Plant Biol.

[CR119] Rojas B, Suárez-Vega F, Saez-Aguayo S, Olmedo P, Zepeda B, Delgado-Rioseco J, Defilippi BG, Pedreschi R, Meneses C, Pérez-Donoso AG, Campos-Vargas R (2021). Pre-anthesis cytokinin applications increase table grape berry firmness by modulating cell wall polysaccharides. Plants.

[CR120] Ruiz-May E, Rose JKC, Seymour G, Poole M, Giovannoni J, Tucker GA (2013). Cell wall architecture and metabolism in ripening fruit and the complex relationship with softening. The molecular biology and biochemistry of fruit ripening.

[CR121] Savoi S, Herrera JC, Forneck A, Griesser M (2019). Transcriptomics of the grape berry shrivel ripening disorder. Plant Mol Biol.

[CR122] Savoi S, Supapvanich S, Hildebrand H, Stralis-Pavese N, Forneck A, Kreil DP, Griesser M (2022). Expression analyses in the rachis hint towards major cell wall modifications in grape clusters showing berry shrivel symptoms. Plants.

[CR123] Schlosser J, Olsson N, Weis M, Reid K, Peng F, Lund S, Bowen P (2008). Cellular expansion and gene expression in the developing grape (*Vitis vinifera* L.). Protoplasma.

[CR124] Schuetz M, Smith R, Ellis B (2013). Xylem tissue specification, patterning, and differentiation mechanisms. J Exp Bot.

[CR125] Schultz HR (2003). Differences in hydraulic architecture account for near-isohydric and anisohydric behaviour of two field-grown *Vitis vinifera* L. cultivars during drought. Plant Cell Environ.

[CR127] Silva CJ, Van Den Abeele C, Ortega-Salazar I, Papin V, Adaskaveg JA, Wang D, Casteel CL, Seymour GB, Blanco-Ulate B (2021). Host susceptibility factors render ripe tomato fruit vulnerable to fungal disease despite active immune responses. J Exp Bot.

[CR129] Swaminathan S, Lionetti V, Zabotina OA (2022). Plant cell wall integrity perturbations and priming for defense. Plants.

[CR130] Tian B, Harrison R, Morton J, Jaspers M (2019). Changes in pathogenesis-related proteins and phenolics in *Vitis vinifera* L. Cv. ‘Sauvignon Blanc’ grape skin and pulp during ripening. Sci Hortic (Amsterdam).

[CR131] Uluisik S, Chapman NH, Smith R, Poole M, Adams G, Gillis RB, Besong TMD, Sheldon J, Stiegelmeyer S, Perez L, Samsulrizal N, Wang D, Fisk ID, Yang N, Baxter C, Rickett D, Fray R, Blanco-Ulate B, Powell ALT, Harding SE, Craigon J, Rose JKC, Fich EA, Sun L, Domozych DS, Fraser PD, Tucker GA, Grierson D, Seymour GB (2016). Genetic improvement of tomato by targeted control of fruit softening. Nat Biotechnol.

[CR132] Vaahtera L, Schulz J, Hamann T (2019). Cell wall integrity maintenance during plant development and interaction with the environment. Nat Plants.

[CR133] Vanholme R, Demedts B, Morreel K, Ralph J, Boerjan W (2010). Lignin Biosynthesis and structure. Plant Physiol.

[CR134] Vicens A, Fournand D, Williams P, Sidhoum L, Moutounet M, Doco T (2009). Changes in polysaccharide and protein composition of cell walls in grape berry skin (cv. Shiraz) during ripening and over-ripening. J Agric Food Chem.

[CR136] Vivier M, Pretorius I (2000). Genetic improvement of grapevine: tailoring grape varieties for the third millennium— a review. South Afr J Enol Vitic.

[CR137] Wang D, Samsulrizal NH, Yan C, Allcock NS, Craigon J, Blanco-Ulate B, Ortega-Salazar I, Marcus SE, Bagheri HM, Perez-Fons L, Fraser PD, Foster T, Fray R, Paul Knox J, Seymour GB (2019). Characterization of CRISPR mutants targeting genes modulating pectin degradation in ripening tomato 1[OPEN]. Plant Physiol.

[CR138] Wang D, Lu Q, Wang X, Ling H, Huang N (2023). Elucidating the role of *SlXTH5* in tomato fruit softening. Hortic Plant J.

[CR139] Weiller F, Schückel J, Willats WGT, Driouich A, Vivier MA, Moore JP (2021). Tracking cell wall changes in wine and table grapes undergoing *Botrytis cinerea* infection using glycan microarrays. Ann Bot.

[CR140] Williamson B, Tudzynski B, Tudzynski P, Van Kan JAL (2007). *Botrytis*
*cinerea*: the cause of grey mould disease. Mol Plant Pathol.

[CR141] Witasari LD, Huang FC, Hoffmann T, Rozhon W, Fry SC, Schwab W (2019). Higher expression of the strawberry xyloglucan endotransglucosylase/hydrolase genes *FvXTH9* and *FvXTH6* accelerates fruit ripening. Plant J.

[CR142] Xu Z, Dai J, Kang T, Shah K, Li Q, Liu K, Xing L, Ma J, Zhang D, Zhao C (2022). *PpePL1* and *PpePL15* are the core members of the pectate lyase gene family involved in peach fruit ripening and softening. Front Plant Sci.

[CR143] Xue C, Guan SC, Chen JQ, Wen CJ, Cai JF, Chen X (2020). Genome wide identification and functional characterization of strawberry pectin methylesterases related to fruit softening. BMC Plant Biol.

[CR144] Yang L, Wang CC, Guo WD, Li XB, Lu M, Yu CL (2006). Differential expression of cell wall related genes in the elongation zone of rice roots under water deficit. Russ J Plant Physiol.

[CR145] Yang L, Huang W, Xiong F, Xian Z, Su D, Ren M, Li Z (2017). Silencing of *SlPL*, which encodes a pectate lyase in tomato, confers enhanced fruit firmness, prolonged shelf-life and reduced susceptibility to grey mould. Plant Biotechnol J.

[CR146] Yi H, Rui Y, Kandemir B, Wang JZ, Anderson CT, Anderson CT (2018). Mechanical effects of cellulose, xyloglucan, and pectins on stomatal guard cells of *Arabidopsis thaliana*. Front Plant Sci.

[CR147] Yıldırım K, Yağcı A, Sucu S, Tunç S (2018). Responses of grapevine rootstocks to drought through altered root system architecture and root transcriptomic regulations. Plant Physiol Biochem.

[CR148] Youssef SM, Amaya I, López-Aranda JM, Sesmero R, Valpuesta V, Casadoro G, Blanco-Portales R, Pliego-Alfaro F, Quesada MA, Mercado JA, Youssef SM, Valpuesta ÁV, Pliego-Alfaro ÁF, Quesada MA, Mercado JA, Casadoro G, Blanco-Portales R (2013). Effect of simultaneous down-regulation of pectate lyase and endo-b-1,4-glucanase genes on strawberry fruit softening. Mol Breed.

[CR149] Yu J, Wang R, Ma W, Lei S, Zhu M, Yang G (2023). Pectate lyase gene VvPL1 plays a role in fruit cracking of table grapes. J Agric Food Chem.

[CR150] Zenoni S, Ferrarini A, Giacomelli E, Xumerle L, Fasoli M, Malerba G, Bellin D, Pezzotti M, Delledonne M (2010). Characterization of transcriptional complexity during berry development in *Vitis vinifera* using RNA-Seq. Plant Physiol.

[CR151] Zhang W, Zhao S, Gu S, Cao X, Zhang Y, Niu J, Liu L, Li A, Jia W, Qi B, Xing Y (2022). *FvWRKY48* binds to the pectate lyase FvPLA promoter to control fruit softening in *Fragaria vesca*. Plant Physiol.

[CR152] Zocca F, Lomolino G, Curioni A, Spettoli P, Lante A (2007). Detection of pectinmethylesterase activity in presence of methanol during grape pomace storage. Food Chem.

