# Supplementary material for: Insights into the cell-wall dynamics in grapevine berries during ripening and in response to biotic and abiotic stresses
Source: Plant Mol Biol. 2024 Apr 11;114(3):38. doi: 10.1007/s11103-024-01437-w (PMC11009762; doi:10.1007/s11103-024-01437-w)
Supplement: Supplementary file 2 — Online Resource 2. Expression heatmaps of the eight major classes of grapevine CWME-encoding genes in 12 contrasts of the grapevine gene expression compendium VESPUCCI (Moretto et al. 2022). Each column of the heatmap corresponds to a contrast comparing B. cinerea infected vs. control samples. The accession numbers of the experiments retrieved by GEO (http://www.ncbi.nlm.nih.gov/geo/) and SRA (https://www.ncbi.nlm.nih.gov/sra) are as follows: 1. GSE52586; 2. PRJNA414966; 3. GSE65969; 4. PRJNA281236. The color scale indicates the log2 expression ratio of each test (infected sample) vs the reference condition (control sample) within each experiment. Genes are considered differentially expressed if they meet a p-value of< 0.01 and an absolute log2 fold change (FC) value ≥ 1.0). E-L = Modified E-L (Eichhorn and Lorenz) grapevine growth stage system from Coombe, B.G. (1995). A selection of the heatmaps represented here is included in Fig. 3, which highlights the genes showing the most interesting expression profiles in the selected contrasts. The heatmaps were generated using RStudio (ver 2023.09.1+494, R ver 4.3.2). Supplementary material 2 (PDF 591.8 kb) [file 11103_2024_1437_MOESM2_ESM.pdf]

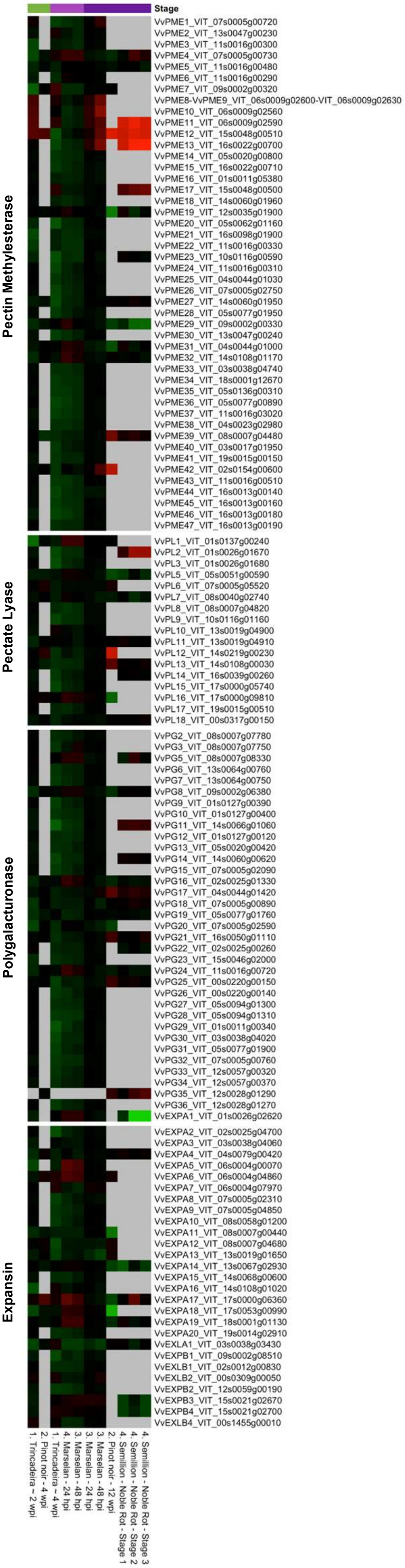

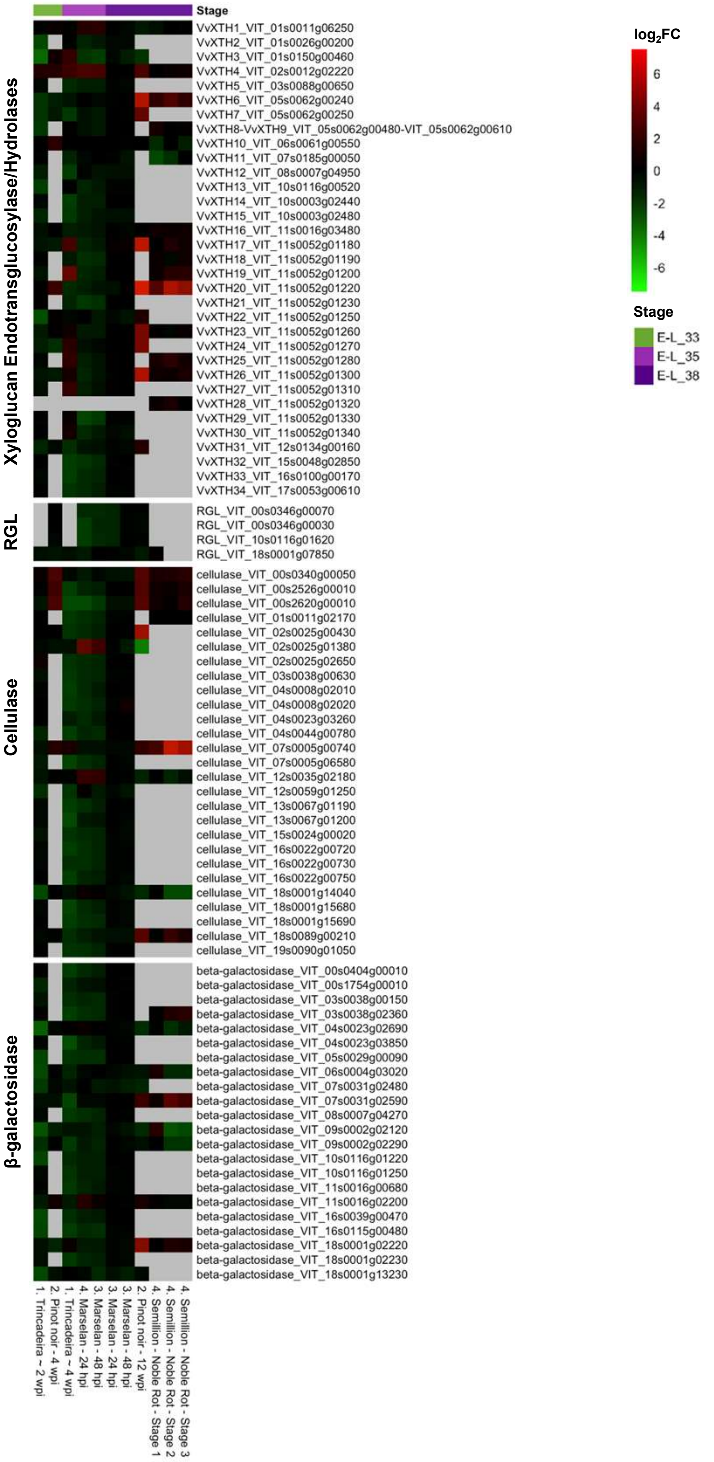

Online Resource 2. Expression heatmaps of the eight most important classes of grapevine CWME encoding genes in 12 contrasts of the grapevine gene expression compendium VESPUCCI (Moretto et al. 2022). Each column of the heatmap corresponds to a contrast comparing B. cinerea infected vs. control samples. The accession numbers of the experiments retrieved by GEO (http://www.ncbi.nlm.nih.gov/geo/) and SRA (https://www.ncbi.nlm.nih.gov/sra) are the following: 1. GSE52586; 2. PRJNA414966; 3. GSE65969; 4. PRJNA281236. The color scale indicates the log2 expression ratio of each test (infected sample) vs. the reference condition (control sample) within each experiment. The genes are considered differentially expressed if they fulfill a p-value of< 0.01 and an absolute log2 Fold Change (FC) value ≥ 1.0). E-L= Modified E-L (Eichhorn and Lorenz) system for grapevine growth stages from Coombe, B.G. (1995). A selection of the heatmaps here represented is included in Fig. 4, which highlights the genes showing the most interesting expression profiles in the selected contrasts. Heatmaps were generated in RStudio (ver 2023.09.1+494, R ver 4.3.2).

*Moretto M, Sonego P, Pilati S, Matus JT, Costantini L, Malacarne G and Engelen K (2022) A COMPASS for VESPUCCI: A FAIR Way to Explore the Grapevine Transcriptomic Landscape. Front. Plant Sci. 13:815443. doi: 10.3389/fpls.2022.815443.

Insights into the cell-wall dynamics in grapevine berries during ripening and in response to biotic and abiotic stresses, *Plant Mol Biol*

Giulia Malacarne+, Jorge Lagreze, Barbara Rojas San Martin, Mickael Malnoy, Marco Moretto, Claudio Moser, Lorenza Dalla Costa
+ correspondence to giulia.malacarne@fmach.it, Research and Innovation Centre, Fondazione Edmund Mach, 38098 San Michele all'Adige, Trento, Italy
