# Supplementary figures and images for: Insights into the cell-wall dynamics in grapevine berries during ripening and in response to biotic and abiotic stresses
Source: Plant Mol Biol. 2024 Apr 11;114(3):38. doi: 10.1007/s11103-024-01437-w (PMC11009762; doi:10.1007/s11103-024-01437-w)

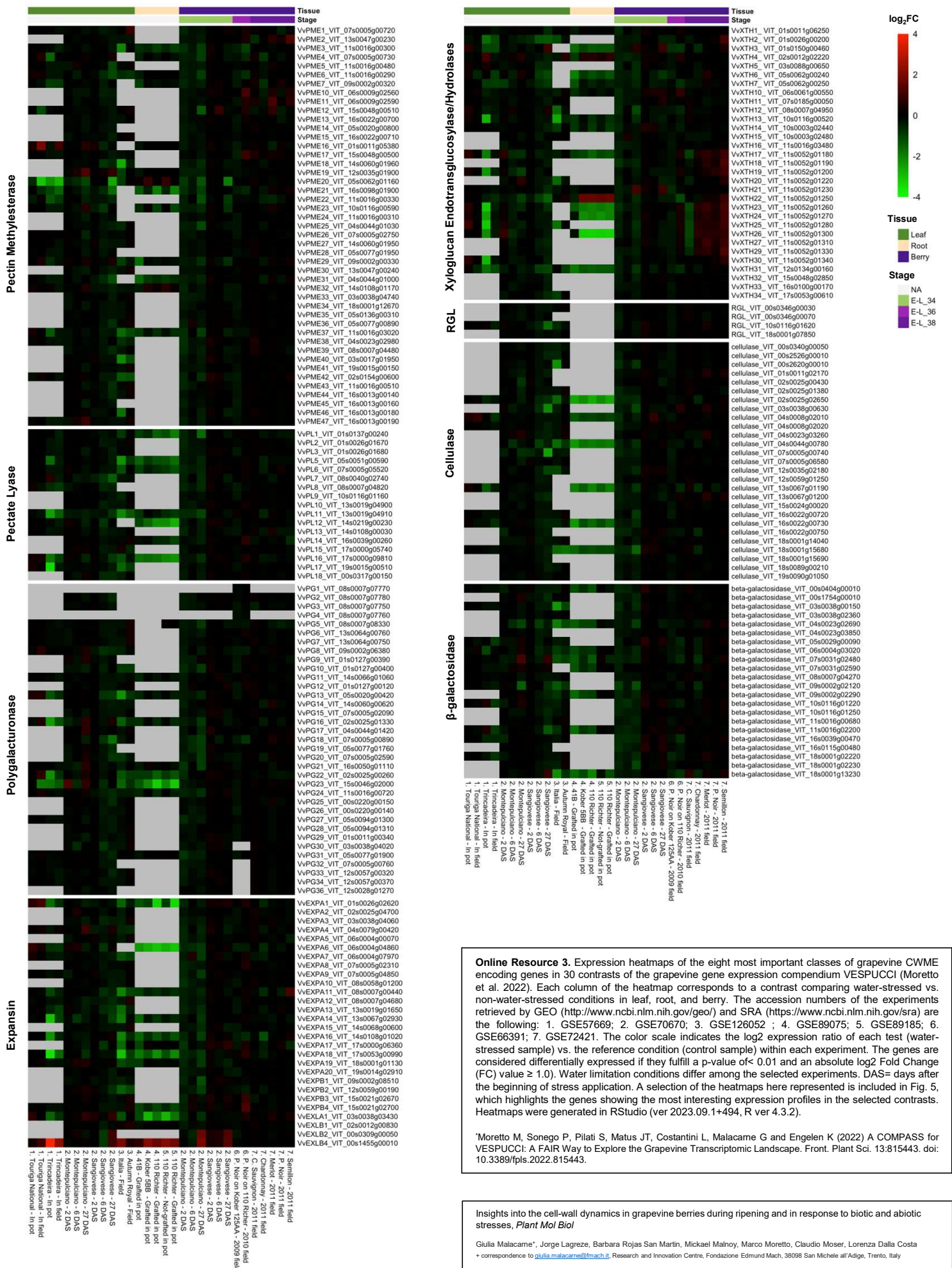

Supplement: Supplementary file 3 — Online Resource 3. Expression heatmaps of the eight major classes of grapevine CWME-encoding genes in 30 contrasts from the grapevine gene expression compendium VESPUCCI (Moretto et al. 2022). Each column of the heatmap corresponds to a contrast comparing water-stressed vs. non-water-stressed conditions in leaf, root, and berry. The accession numbers of the experiments retrieved from GEO (http://www.ncbi.nlm.nih.gov/geo/) and SRA (https://www.ncbi.nlm.nih.gov/sra) are as follows: 1. GSE57669; 2. GSE70670; 3. GSE126052; 4. GSE89075; 5. GSE89185; 6. GSE66391; 7. GSE72421. The color scale indicates the log2 expression ratio of each test (water-stressed sample) versus. the reference condition (control sample) within each experiment. Genes are considered differentially expressed if they meet a p-value of< 0.01 and an absolute log2 fold change (FC) value ≥ 1.0). Water restriction conditions varied among the selected experiments. DAS= days after start of stress application. A selection of the heatmaps shown here is included in Fig. 4, which highlights the genes showing the most interesting expression profiles in the selected contrasts. The heatmaps were generated using RStudio (ver 2023.09.1+494, R ver 4.3.2). Supplementary material 3 (PDF 655.3 kb) [file 11103_2024_1437_MOESM3_ESM.pdf]
